# 5-methoxytryptamine improves hepatic inflammation and insulin resistance in a macrophage C-X-C motif chemokine ligand 14 dependent manner

**DOI:** 10.1186/s43556-026-00507-3

**Published:** 2026-06-28

**Authors:** Xiaoyu Liao, Yuxi Xiao, Bingyao Liu, Yuan Dong, Hang Yang, Linlin Zhang, Dong Li, Peiye Sun, Yixiang Feng, Ying Yang, Liqun Zhang, Qingwu Yang, Qiang Tong, Hongting Zheng

**Affiliations:** 1 Department of Endocrinology, Metabolic and Chronic Disease Science Innovation Center, Translational Research of Diabetes Key Laboratory of Chongqing, The Second Affiliated Hospital of Army Medical University, Chongqing, 400037 China; 2 Department of General Medicine, General Hospital of Western Theater Command, Chengdu, Sichuan 610083 China; 3 Department of Endocrinology, General Hospital of Western Theater Command, Chengdu, Sichuan 610083 China; 4 Department of Endocrinology, Chongqing Hospital of Traditional Chinese Medicine, Chongqing, 400021 China; 5 Department of Clinical Laboratory Medicine, The Second Affiliated Hospital of Army Medical University, Chongqing, 400037 China; 6 Department of Neurology, The Second Affiliated Hospital of Army Medical University, Chongqing, 400037 China

**Keywords:** 5-methoxytryptamine, Insulin resistance, Inflammation, CXCL14, Macrophages

## Abstract

Accumulating evidence indicates that tryptophan metabolism plays important roles in insulin resistance, and glucolipid metabolism. Our preliminary study revealed a significant decrease in the serum levels of the tryptophan metabolite 5-methoxytryptamine (5MT), in both high-fat diet (HFD)-fed and db/db mice. Notably, these levels were restored following treatment with the hypoglycemic agent, the dipeptidyl peptidase-4 inhibitor (DPP-4i) sitagliptin, suggesting a potential relationship between 5MT and metabolic homeostasis. In the current study, we demonstrated that 5MT treatment ameliorated insulin resistance and hepatic steatosis in HFD and db/db mice. Specific inhibition of the aryl hydrocarbon receptor (AHR) attenuated the beneficial effects of 5MT on insulin resistance in HFD mice. Transcriptome sequencing and subsequent validation revealed that 5MT significantly upregulated the expression of C-X-C motif chemokine ligand 14 (*Cxcl14*) in hepatic macrophages. Myeloid-specific knockout of *Cxcl14* demonstrated that 5MT inhibited hepatic inflammatory response to improve insulin resistance in a macrophage CXCL14-dependent manner. Furthermore, molecular mechanism exploration revealed that 5MT promoted the AHR-mediated transcriptional activation of *Cxcl14*, which inhibited glycolysis in macrophages, subsequently suppressing their proinflammatory classical activation (M1 phenotype). Collectively, these data reveal a novel beneficial role of 5MT in insulin resistance and metabolic homeostasis, providing a rationale for new therapeutic strategies targeting insulin resistance-associated metabolic disorders, such as obesity and diabetes.

**Supplementary Information:**

The online version contains supplementary material available at 10.1186/s43556-026-00507-3.

## Introduction

Insulin resistance is a pivotal pathological hallmark underlying various metabolic disorders, including obesity and type 2 diabetes mellitus (T2DM) [1, 2]. The mechanism of insulin resistance is intricate and not yet completely understood; therefore, further revealing its pathogenesis holds promise for exploring novel targets to promote insulin sensitivity. As a microenvironmental factor, dysbiosis of the gut microbiota has been closely implicated in the onset and progression of insulin resistance, obesity, diabetes, etc. [3, 4]. Accumulating evidence indicates that the metabolites produced by gut bacteria are crucial mediators of host-microbial crosstalk. Metabolites such as short-chain fatty acids (SCFAs), bile acids (BAs) and tryptophan [5–7] have been shown to modulate intestinal barrier integrity as well as inflammatory response, insulin resistance and glucolipid metabolism. Consequently, restoring the gut microbiota or supplementation with specific metabolites may represent a promising therapeutic strategy to improve insulin resistance and reestablish metabolic homeostasis.

In humans, tryptophan is an essential amino acid obtained exclusively from dietary intake; it plays a crucial role in protein synthesis and serves as a precursor for the biosynthesis of important bioactive compounds, such as serotonin and melatonin [8]. In addition to the serotonin and kynurenine pathways, tryptophan can also be metabolized by gut bacteria (e.g., Bacteroides) to produce a variety of indole derivatives [9]. Emerging evidence suggests that tryptophan metabolism affects diverse physiological and pathological processes, including neural function, immune and inflammatory responses, and metabolic regulation [10–12]. The circulating level of tryptophan has been found to be positively correlated with body mass index (BMI) and homeostatic model assessment for insulin resistance (HOMA-IR), and positively associated with the risk of T2DM [13]. In addition, tryptophan metabolism may shift from the host kynurenine pathway towards the production of indole derivatives by gut bacteria in mice with a genetic deficiency of indoleamine 2,3-dioxygenase, potentially contributing to the improvement in insulin sensitivity [14]. These studies indicate that tryptophan metabolism plays a pivotal role in regulating insulin resistance and metabolic homeostasis.

Tryptophan metabolites have been identified as ligands for the aryl hydrocarbon receptor (AHR) [15, 16]. For instance, indole-3-lactic acid (ILA), a tryptophan derivative, has been reported to repress nuclear factor-κB (NF-κB) signaling by activating AHR, and reduce the accumulation of inflammatory macrophages, thereby alleviating colitis [17]. The activation of AHR is involved in various biological processes, including intestinal permeability, mucosal inflammation, incretin secretion, and insulin resistance, exerting beneficial effects against metabolic diseases [18].

In a previous study, we demonstrated that changes in the gut microbiota induced by the hypoglycemic agent dipeptidyl peptidase-4 inhibitor (DPP-4i) sitagliptin (Sit) improved glucose homeostasis in high-fat diet (HFD)-fed mice, which may be mediated by metabolite alterations [19]. An in-depth analysis of untargeted metabolomics data revealed that the levels of approximately 82% of amino acids, including tryptophan and branched-chain amino acids, significantly changed. Notably, the level of 5-methoxytryptamine (5MT) showed the highest increase in DPP-4i treated HFD mice compared with control mice (810 versus 0, relative content). 5MT, also referred to as 5-methoxyindole-3-ethylamine, is a derivative produced by tryptophan metabolism [20], and has been reported to scavenge free radicals and radical products, and act as an antioxidant against reactive oxygen species [21]. In preliminary results from the present study, targeted metabolite detection using liquid chromatography-tandem mass spectrometry (LC–MS/MS) analysis revealed that the serum concentrations of 5MT in HFD and db/db mice were obviously lower than those in control mice, and increased after DPP-4i Sit treatment, a finding that was similar to the results for fecal samples from HFD mice in our previous study [19]. These preliminary results suggest that the metabolite 5MT may be associated with metabolic homeostasis in obese and diabetic mice. Nevertheless, the function of 5MT in metabolic regulation has not yet been established, which constitutes the primary objective of our study.

In the present study, we investigated the role of 5MT in improving insulin resistance and hepatic steatosis, and revealed that the effects of 5MT are dependent on AHR activation and the subsequent upregulation of the expression of C-X-C motif chemokine ligand 14 (CXCL14) in macrophages. Furthermore, we explored the mechanism by which 5MT ameliorates insulin resistance by inhibiting hepatic inflammation through the CXCL14-mediated suppression of classical macrophage activation. These findings identify 5MT as a promising therapeutic target for insulin resistance-associated metabolic diseases.

## Results

### 5MT treatment improves insulin resistance and hepatic steatosis in HFD and db/db mice

In a preliminary study, we measured serum 5MT levels in mouse models of obesity (HFD) and T2DM (db/db). As shown in Fig. S1a, b, compared with those in control mice, serum 5MT levels were significantly lower in HFD and db/db mice. After treatment with DPP-4i Sit, 5MT levels significantly recovered; the trend of change was similar to that observed for fecal samples from HFD mice [19]. These results suggest that the metabolite 5MT may be related to metabolic homeostasis in obese and T2DM mice.

To determine the appropriate dose of 5MT in vivo, 8–9 weeks old C57BL/6 J mice were orally administered 5MT at doses of 5, 10, and 30 mg/kg daily for 6 weeks on the basis of the reported experimental dose of tryptophan derivatives [22, 23]. No side effects were observed in the liver or kidney tissues of C57BL/6 J mice treated with different doses of 5MT (Fig. S2a-e). Moreover, following 5MT treatment, the 5MT levels in the liver were greater than those in the muscle and adipose tissue in C57BL/6 J mice (Fig. S2f). Therefore, in this study, we administered 5MT at a dosage of 30 mg/kg/day to investigate the effects of 5MT on metabolism.

**Fig. 1:**
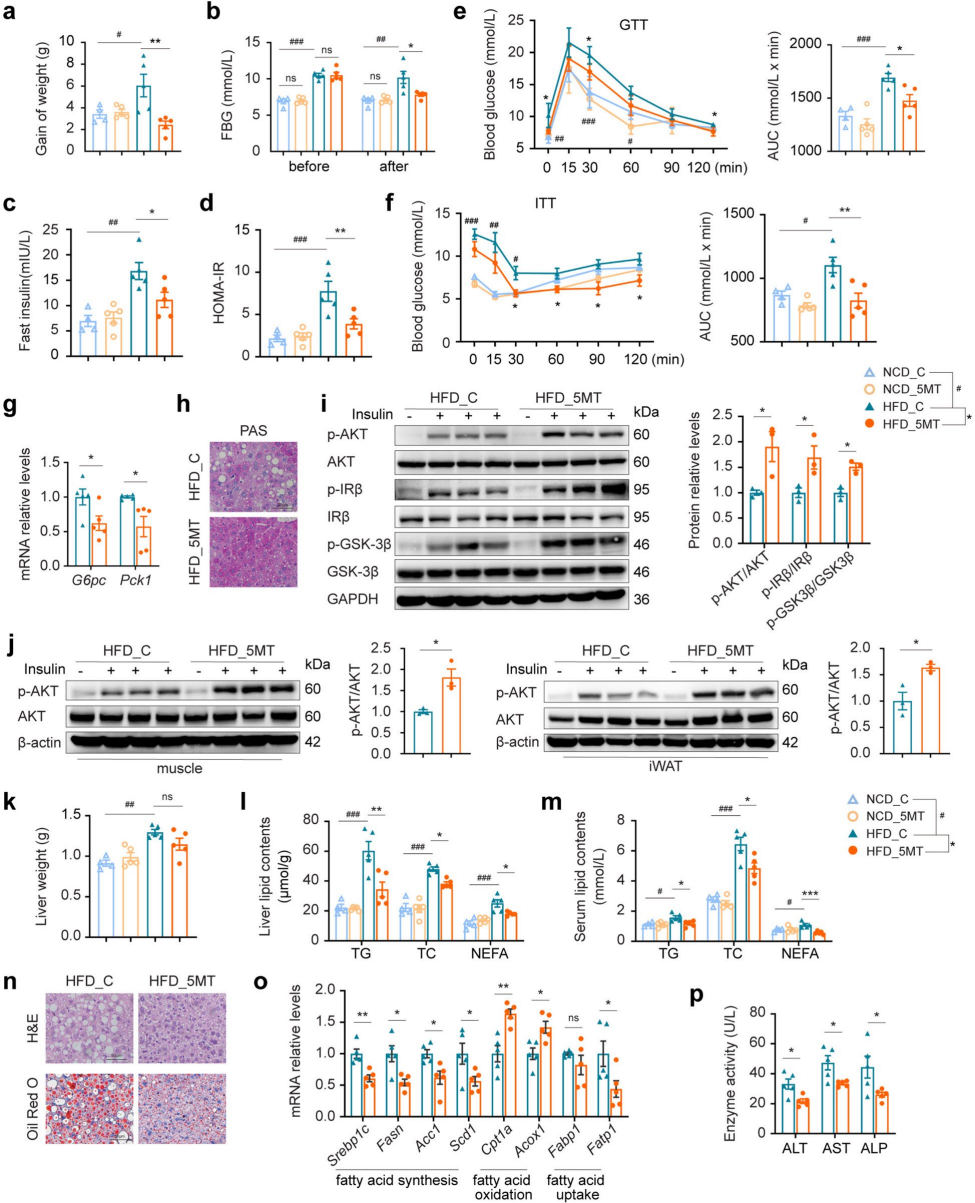
5MT improves HFD- induced insulin resistance and hepatic steatosis. NCD and HFD mice were treated with 5MT (30 mg/kg) or vehicle for 6 weeks. **a**, **b** Body weight gain and FBG levels of NCD and HFD mice treated with or without 5MT. **c**, **d** Fasting serum insulin levels and the HOMA-IR index. **e**, **f** Blood glucose levels during the GTT and ITT and the corresponding areas under the curves. **g** mRNA expression levels of *G6pc* and *Pck1* in liver samples from the indicated HFD mice. **h** Representative images of PAS-stained liver sections. **i**, **j** Expression levels of phosphorylated and total AKT, IRβ and GSK-3β in the liver (**i**), muscle and iWAT (**j**) of HFD_C and HFD_5MT mice. GAPDH and β-actin were used as loading controls. **k** Liver weights of NCD and HFD mice treated with or without 5MT. **l**, **m** TG, TC and NEFA contents in the liver (**l**) and serum (**m**). **n** Representative images of H&E- and oil red O-stained liver sections. **o** mRNA levels of genes regulating fatty acid synthesis, oxidation and uptake in the liver. **p** Liver function assessment via serum ALT, AST, and ALP levels. Scale bar, 50 μm. N = 4–5 mice per group; all data in bar plots are expressed as the mean ± S.E.M. ^#^*P* < 0.05, ^##^*P* < 0.01, and ^###^*P* < 0.001 versus the NCD_C group; ^*^P < 0.05, ^**^P < 0.01 and ^***^P < 0.001 versus the HFD_C group; ns, no significance

Normal chow diet (NCD) or HFD mice were administered 5MT or vehicle for the last 6 weeks of the feeding period. As expected, compared with the NCD mice, the HFD mice exhibited greater body weight and impaired glucose tolerance and insulin sensitivity (Fig. 1a-f). During the treatment period, the body weight of 5MT-treated HFD mice (HFD_5MT) was lower than that of control mice (HFD_C), although the difference was not significant for each week (Fig. 1a and Fig. S3a). Moreover, no statistically significant differences in lean mass or fat mass were detected between the two groups. (Fig. S3b). After 5MT treatment, the fasting blood glucose (FBG) levels, fasting insulin levels, and HOMA-IR of HFD mice were significantly decreased (Fig. 1b-d). Additionally, compared with HFD_C mice, HFD_5MT mice exhibited ameliorated glucose intolerance and insulin resistance (Fig. 1e, f). Moreover, 5MT treatment decreased the mRNA expression levels of gluconeogenic genes (*G6pc* and *Pck1*), and increased the glycogen content in the livers of HFD mice (Fig. 1g, h). As shown in Fig. 1i, in HFD mice after 5MT treatment, the expression of phosphorylated IRβ, AKT and GSK3β was upregulated in the liver, and similar results were observed in the muscle and subcutaneous white adipose tissue (iWAT) (Fig. 1j). Taken together, these results indicate that 5MT treatment improved HFD-induced insulin resistance.

In addition, given that hepatic steatosis is closely related to insulin resistance and serves as a hallmark feature of HFD mice, we also examined the effect of 5MT on hepatic lipid metabolism. Compared with HFD_C mice, HFD_5MT mice did not exhibit a significant change in liver weight (Fig. 1k), but hepatic and serum triglyceride (TG), total cholesterol (TC) and nonesterified fatty acid (NEFA) levels were significantly reduced (Fig. 1l, m). Consistent with the alterations in hepatic lipid content, fewer lipid droplets were observed in the livers of HFD mice treated with 5MT (Fig. 1n). Similarly, the mRNA expression levels of genes related to fatty acid synthesis (*Srebp1c*, *Fasn*, *Acc1*, and *Scd1*) and fatty acid uptake (*Fatp1*) were significantly decreased after 5MT treatment. In contrast, the mRNA expression levels of genes involved in fatty acid β-oxidation (*Cpt1a* and *Acox1*) were increased in HFD_5MT mice (Fig. 1o). In addition, the liver function of HFD_5MT mice improved, as indicated by decreases in serum alanine aminotransferase (ALT), aspartate aminotransferase (AST) and alkaline phosphatase (ALP) concentrations (Fig. 1p), further confirming the protective effect of 5MT against hepatic steatosis.

**Fig. 2:**
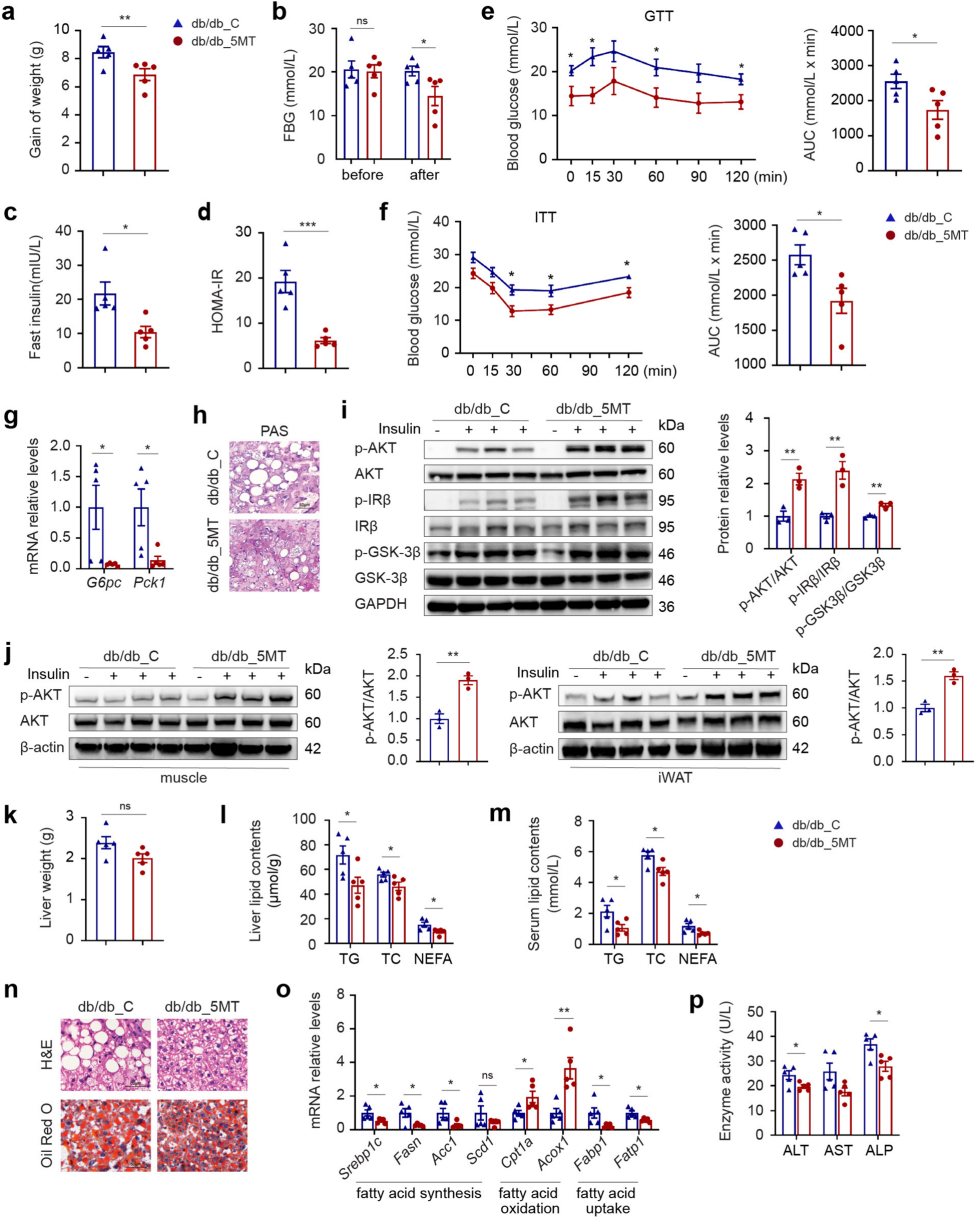
5MT ameliorates gene deficiency-induced insulin resistance and hepatic steatosis in db/db mice. db/db mice were treated with 5MT (30 mg/kg) or vehicle for 6 weeks. **a, b** Body weight gain and FBG levels of db/db mice treated with or without 5MT. **c**, **d** Fasting serum insulin levels and the HOMA-IR index. **e**, **f** Blood glucose levels during the GTT (**e**) and ITT (**f**) and the corresponding areas under the curves. **g** mRNA expression levels of *G6pc* and *Pck1* in liver samples. **h** Representative images of PAS-stained liver sections. **i**, **j** Expression levels of phosphorylated and total AKT, IRβ and GSK-3β in the liver (**i**), muscle and iWAT (**j**). GAPDH and β-actin were used as loading controls. **k** Liver weights of db/db mice treated with or without 5MT. **l**, **m** TG, TC and NEFA contents in the liver (**l**) and serum (**m**). **n** Representative images of H&E- and oil red O-stained liver sections. **o** mRNA levels of genes regulating fatty acid synthesis, oxidation and uptake in the liver. **p** Liver function assessment via serum ALT, AST, and ALP levels. Scale bar, 50 μm. n = 5 mice per group; all data in bar plots are expressed as the mean ± S.E.M. ^*^P < 0.05, ^**^P < 0.01 and ^***^P < 0.001 versus the db/db_C group; ns, no significance

Furthermore, in db/db mice, 5MT treatment significantly decreased body weight gain, FBG levels, and fasting insulin levels (Fig. 2a-c and Fig. S3c). Moreover, after 5MT treatment, glucose intolerance and insulin resistance were improved, and hepatic gluconeogenesis was decreased (Fig. 2d-h). The insulin signaling pathway was also activated in 5MT-treated db/db mice (Fig. 2i, j). Additionally, in db/db_5MT mice, hepatic and serum lipid accumulation was markedly reduced (Fig. 2k-n), with inhibition of fatty acid synthesis and increased fatty acid β-oxidation (Fig. 2o). Following 5MT treatment, liver function was also improved in db/db mice (Fig. 2p). These findings substantiate the role of 5MT in alleviating insulin resistance and hepatic steatosis induced by genetic deficiency. Moreover, after 5MT treatment, the expression levels of fatty acid synthesis-related markers were decreased in the iWAT of HFD and db/db mice, accompanied by increased PPARα expression (Fig. S3d-g), suggesting that 5MT also has beneficial effects on lipid metabolism in iWAT, which may be associated with the improvement in insulin resistance.

### 5MT alleviates insulin resistance by activating AHR

**Fig. 3:**
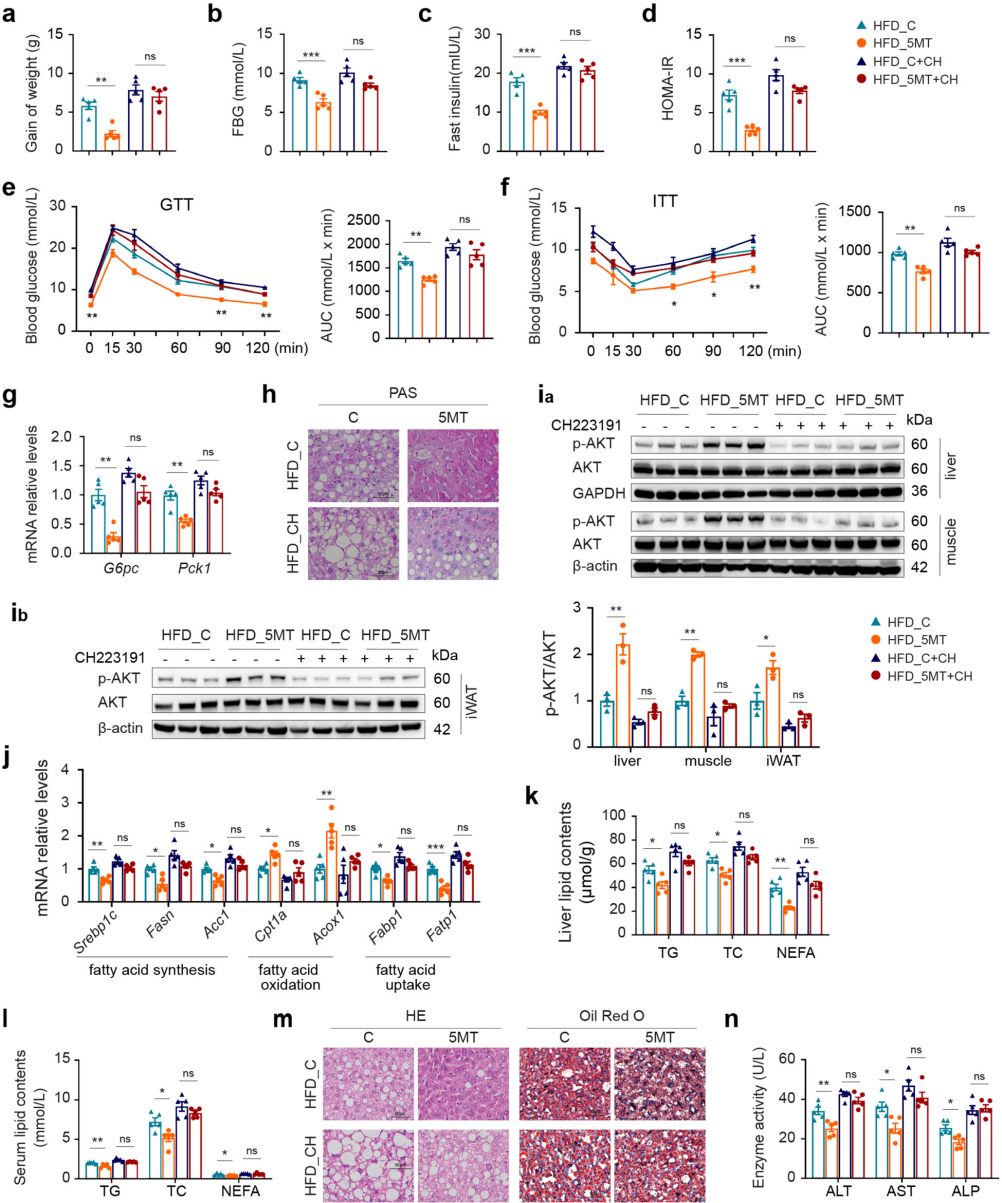
5MT alleviates insulin resistance by activating AHR. To inhibit AHR activity, HFD mice were administered CH-223191 at a dosage of 10 mg/kg for the last 10 days of the 5MT treatment period. **a**, **b** Body weight gain and FBG levels of HFD mice treated with vehicle, 5MT and/or the AHR inhibitor CH223191. **c**, **d** Fasting serum insulin levels and HOMA-IR index in each group of mice. **e**, **f** Blood glucose levels during the GTT and ITT and the corresponding areas under the curves. **g** mRNA expression levels of *G6pc* and *Pck1* in liver samples. **h** Representative images of PAS-stained liver sections. **i** Expression levels of phosphorylated and total AKT in the liver, muscle and iWAT. GAPDH and β-actin were used as loading controls. **j** mRNA levels of genes regulating fatty acid synthesis, oxidation and uptake in the liver. **k**, **l** TG, TC and NEFA contents in the liver (**k**) and serum (**l**). **m** Histological images of H&E- and oil red O- stained liver sections from the indicated mice. **n** ALT, AST, and ALP levels in the serum. Scale bar, 50 μm. n = 5 mice per group; all data in bar plots are expressed as the mean ± S.E.M. ^*^P < 0.05, ^**^P < 0.01 and ^***^P < 0.001 versus the HFD_C group; ns, no significance

Considering that AHR, a ubiquitously expressed ligand-activated transcription factor, likely mediates the role of tryptophan metabolites in metabolic regulation [24, 25], we assessed the expression of AHR target genes. *Cyp1a1* and *Ahrr* expression was significantly upregulated in the livers of 5MT-treated HFD mice (Fig. S4a), which suggests that 5MT might act as a ligand to promote AHR activity. Next, we examined the extent to which AHR inhibition alters the effect of 5MT on insulin resistance. CH223191, a specific AHR antagonist, exhibited no significant effect on glucose tolerance, insulin sensitivity or hepatic lipid metabolism in NCD mice (Fig. S5a-k). As expected, the expression of AHR target genes upregulated by 5MT was decreased in CH223191-treated HFD mice (Fig. S4b). Consistently, the effects of 5MT on decreasing body weight, FBG, and fasting insulin levels were attenuated in HFD mice treated with CH223191 (Fig. 3a-c). The improvement in glucose intolerance and insulin resistance mediated by 5MT was abolished by CH223191 treatment (Fig. 3d-f). Similarly, the inhibition of gluconeogenesis and activation of the insulin pathway in 5MT-treated mice were weakened by the inhibitor CH223191 (Fig. 3g-i). The reduction in fatty acid synthesis and the increase in fatty acid β-oxidation induced by 5MT were also blocked (Fig. 3j). Consequently, CH223191 reversed the role of 5MT in reducing lipid accumulation in the liver and serum (Fig. 3k-m), and improving liver function in HFD mice (Fig. 3n). Taken together, these results suggest that 5MT plays a role in improving insulin resistance and hepatic steatosis by activating the receptor AHR.

### 5MT improves insulin resistance in a macrophage CXCL14-dependent manner

**Fig. 4:**
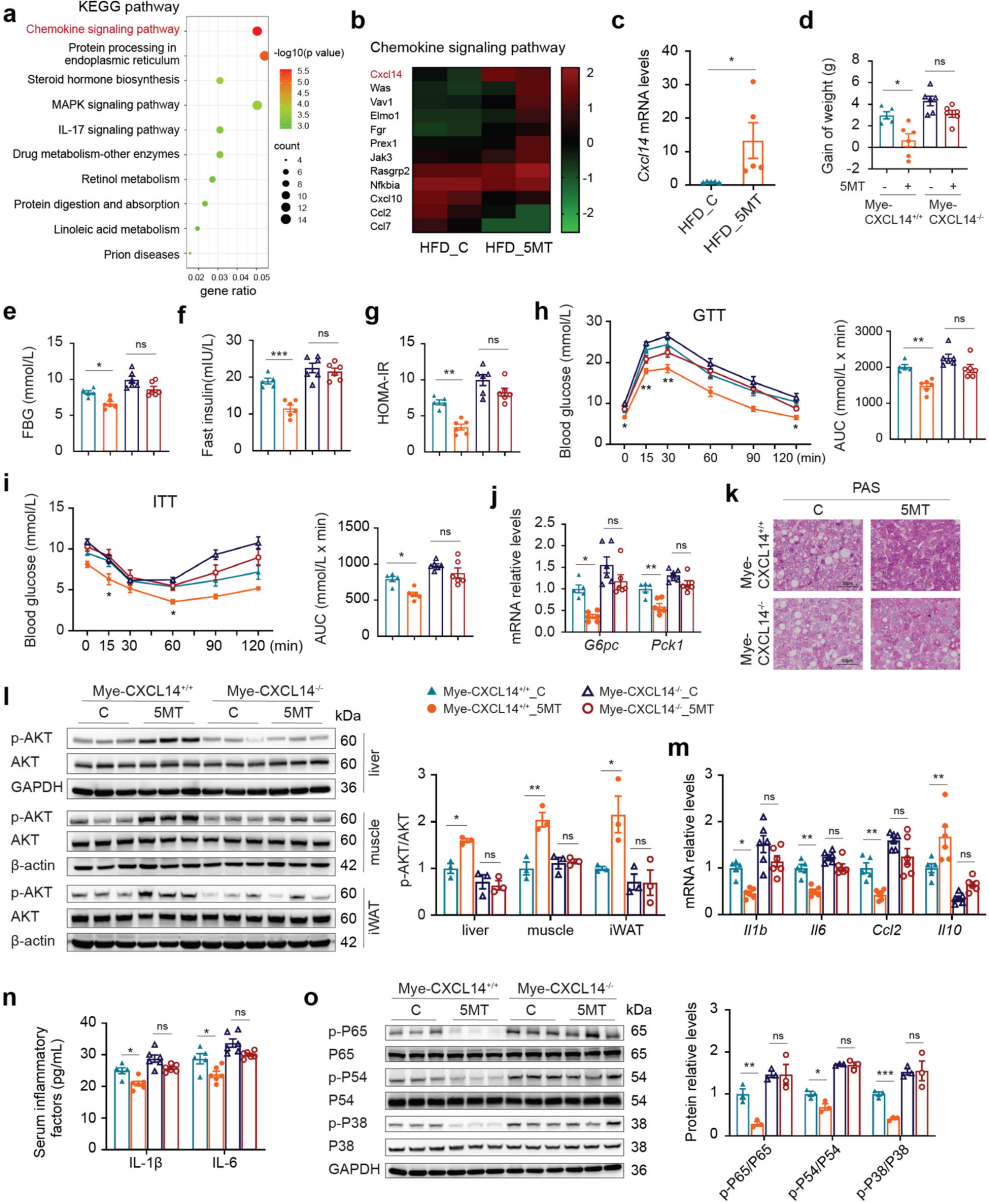
5MT improves insulin resistance in a macrophage Cxcl14-dependent manner. **a**, **b** Total RNA was extracted from the liver tissues of HFD_C and HFD_5MT mice, and RNA sequencing was performed. n = 2 mice per group. **a** KEGG analysis of differentially expressed genes and the 10 highest-ranking signaling pathways. **b **Heatmap showing the genes with significantly differential expression in the “chemokine signaling pathway”. **c** mRNA expression levels of Cxcl14 in the livers of HFD_C and HFD_5MT mice. n = 5 mice per group. **d-o** HFD-fed Mye-CXCL14^+/+^ and Mye-CXCL14^−/−^ mice were treated with 5MT (30 mg/kg, 6 weeks) or vehicle. **d-g** Body weight gain, FBG, fasting serum insulin levels and HOMA-IR index. **h**, **i** Blood glucose levels during the GTT and ITT and the corresponding areas under the curves. **j** mRNA expression levels of *G6pc* and *Pck1* in liver tissues. **k** Representative images of PAS-stained liver sections. Scale bar, 50 μm. **l** Protein expression levels of phosphorylated and total AKT in the liver, muscle and iWAT. GAPDH and β-actin were used as loading controls. **m** mRNA expression levels of *Il1b*, *Il6*, *Ccl2* and *Il10* in the liver. **n** Serum levels of IL-1β and IL-6. **o** Protein expression levels of phosphorylated and total P65, P54 and P38 in the liver. GAPDH was used as the loading control. All data in bar plots are expressed as the mean ± S.E.M. In **d**-**o**, n = 5–6 mice per group. ^*^P < 0.05 and ^**^P < 0.01 versus the Mye-CXCL14^+/+^_C group; ns, no significance

Next, to explore the molecular mechanism underlying the role of 5MT in improving insulin resistance, we performed RNA sequencing analysis of liver tissues from HFD mice treated with or without 5MT. The genes with significantly altered expression levels (Fold change > 1.5) are listed in Table S1. Hierarchical clustering of the top 20 upregulated genes and the top 20 downregulated genes revealed distinct expression patterns between 5MT-treated and control mice (Fig. S6a). Kyoto Encyclopedia of Genes and Genomes (KEGG) pathway analysis revealed that the “chemokine signaling pathway” was strongly regulated by 5MT treatment (Fig. 4a). Gene Ontology (GO) analysis revealed that the biological processes affected by 5MT included the inflammatory response, innate immune response and defense response. (Fig. S6b, c). Chemokines are the main drivers of liver inflammation, and regulate the activation and migration of hepatic macrophages, neutrophils and other cells [26]. Given that chronic inflammation represents one of the most important pathologic factors of insulin resistance and diabetes [27], and considering that tryptophan metabolites act as ligands of AHR to participate in inflammation and immune responses [28], we speculated that 5MT might be involved in regulating the inflammatory response to improve insulin resistance. Thus, we first confirmed that the mRNA levels of *Il1b*, *Il6* and *Ccl2* were obviously decreased, and the level of *Il10* (an anti-inflammatory factor) was increased in the livers of 5MT-treated HFD mice (Fig. S6d). Consistently, the serum levels of proinflammatory cytokines (IL-1β and IL-6) were significantly lower in HFD_5MT mice than in HFD_C mice (Fig. S6e). Moreover, we evaluated the effect of 5MT on inflammation-related NF-κB signaling, and observed that the protein levels of phosphorylated P65, JNK (P54/P46) and P38 were reduced in the livers of HFD_5MT mice compared to the control mice (Fig. S6f). These results suggest that 5MT alleviates the HFD-induced hepatic inflammatory response.

In the “chemokine signaling pathway”, the significantly enriched genes included *Cxcl14*, *Cxcl10*, *Ccl2*, and *Ccl7* (Fig. 4b). Among these genes, *Cxcl14*, a member of the CXC chemokine family, exhibited the most differential expression (p adjusted = 5.33 × 10^–13^), with the highest-fold upregulation (fold change = 49.68). CXCL14 has been reported to suppress macrophage infiltration and inflammation [29, 30]. To validate the RNA sequencing data, we assessed the expression levels of *Cxcl14* in the livers of HFD mice, and the results indicated that *Cxcl14* expression was obviously upregulated by 5MT treatment (Fig. 4c). Owing to the presence of multiple cell types in the liver, to clarify which cell type 5MT mainly acts on, we analyzed the mRNA and protein expression levels of CXCL14 in primary cells isolated from the liver. As shown in Fig. S7a-e, the expression level of CXCL14 was greater in hepatic macrophages than in primary hepatocytes and hepatic stellate cells (HSCs). Moreover, 5MT markedly upregulated the expression of *Cxcl14* in mouse bone marrow-derived macrophages (BMDMs) in a dose-dependent manner (Fig. S7f), but not in hepatocytes or HSCs (Fig. S7g, h). Immune cells, especially liver-residing Kupffer cells and recruited macrophages, have been identified as key regulators of hepatic inflammation [31]. Thus, we investigated whether macrophage CXCL14 mediates the effects of 5MT on hepatic inflammation and insulin resistance in HFD mice.

First, we used the Cre-LoxP system to generate myeloid-specific *Cxcl14*-deficient mice (Mye-CXCL14^−/−^). After genotyping (Fig. S8a), CXCL14 expression levels in different tissues (Fig. S8b-d) and in isolated primary hepatocytes, hepatic macrophages and BMDMs (Fig. S8e-g) were assessed by qPCR and immunoblot analysis, to verify the specific knockout of *Cxcl14*. Then, HFD-fed Mye-CXCL14^+/+^ and Mye-CXCL14^−/−^ mice were treated with or without 5MT. Consistent with the results observed in HFD_5MT and db/db_5MT mice, 5MT clearly improved HFD-induced glucose intolerance and insulin resistance in Mye-CXCL14^+/+^ mice (Fig. 4d-l). However, in Mye-CXCL14^−/−^ mice, 5MT treatment had no significant effects on body weight, fasting insulin levels, HOMA-IR index, and blood glucose levels in the glucose tolerance test (GTT) and insulin tolerance test (ITT) (Fig. 4d-i). The 5MT-mediated inhibition of gluconeogenesis, and activation of the insulin signaling pathway in the livers of Mye-CXCL14^+/+^ mice were also abolished (Fig. 4j-l). Additionally, the myeloid-specific knockout of *Cxcl14* attenuated the ameliorative effect of 5MT on HFD-induced hepatic steatosis (Fig. S9a-g). Moreover, in Mye-CXCL14^−/−^ mice, 5MT treatment did not significantly decrease the levels of proinflammatory factors in the liver or serum (Fig. 4m, n), and did not effectively inhibit the inflammation-associated NF-κB pathway (Fig. 4o). However, the specific knockout of *Cxcl14* did not affect the 5MT-induced upregulation of AHR target gene expression (Fig. S9h). Together, these results demonstrate that macrophage CXCL14 is involved in inhibiting hepatic inflammation to improve insulin resistance mediated by 5MT.

### 5MT inhibits classical macrophage activation by upregulating CXCL14 expression

In a previous study, CXCL14 promoted the M2 polarization of macrophages in adipose tissue [32]. The dysregulation of macrophage polarization is involved in the induction of insulin resistance [33, 34]. Therefore, we explored whether 5MT induced alterations in CXCL14 expression are associated with macrophage activation in the livers of HFD mice. Immunofluorescence labeling of F4/80^+^NOS2^+^ cells confirmed a decrease in M1 type macrophages in the livers after 5MT treatment (Fig. S10a). In addition, this phenotypic change was confirmed by obvious reductions in the expression of M1-related markers (*Nos2* and *Rantes*), and increases in the expression of M2-related markers (*Arg1*, *Clec10a*, *Mgl2* and *Mrc1*) in the livers of HFD_5MT mice (Fig. S10b, c). Moreover, the *Arg1*/*Nos2* ratio, an index of the extent of M2/M1 macrophage polarization, [35], was significantly increased (Fig. S10d).

**Fig. 5:**
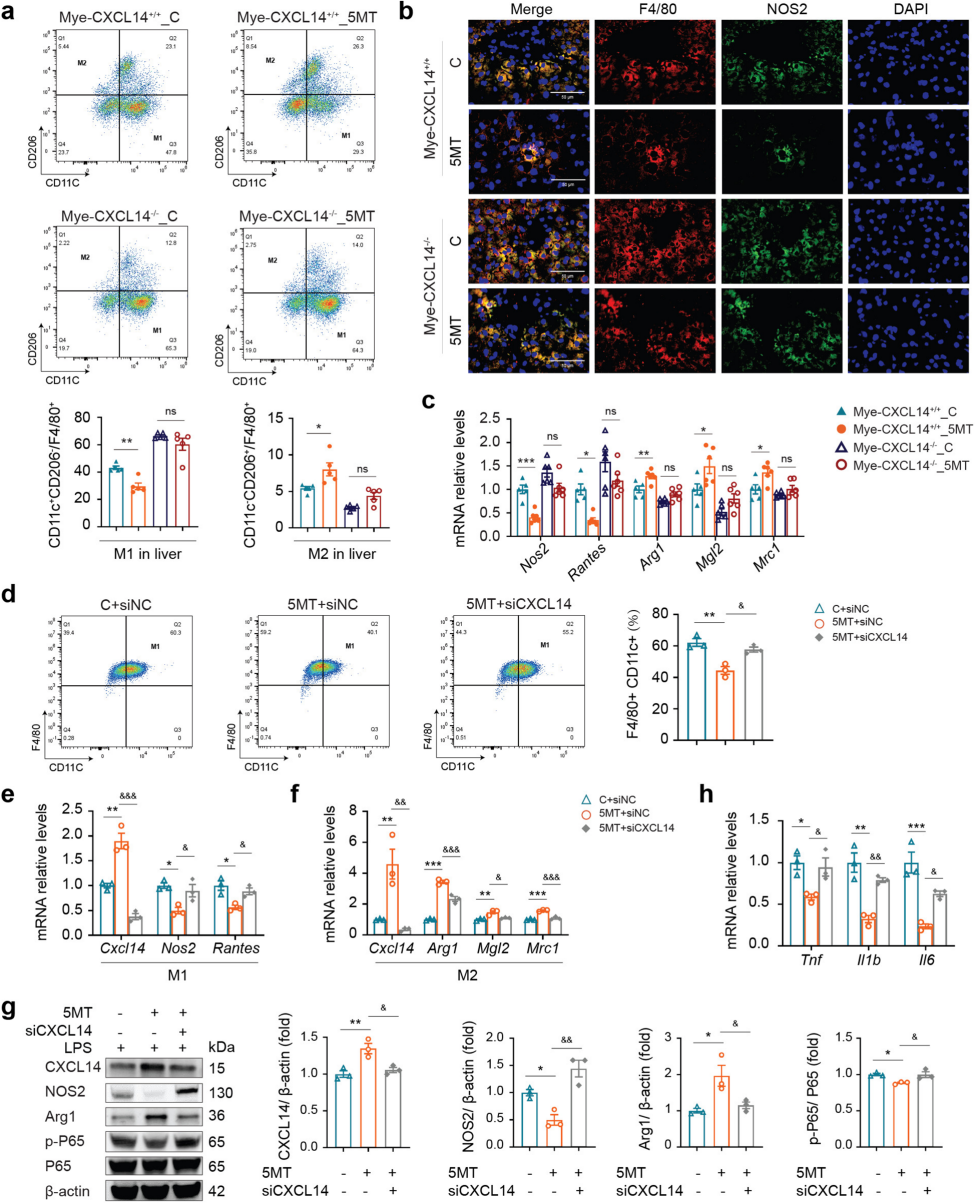
5MT inhibits M1 macrophage polarization by upregulating CXCL14 expression. **a-c** HFD-fed Mye-CXCL14^+/+^ and Mye-CXCL14^−/−^ mice were treated with 5MT or vehicle. **a** Frequencies of M1 (F4/80^+^CD11c^+^ CD206^−^) and M2 (F4/80^+^CD11c^−^ CD206^+^) macrophages in the liver. **b** IF staining of F4/80 and NOS2 in liver tissue sections. Scale bar, 50 μm. **c** mRNA expression levels of M1 (*Nos2* and *Rantes*) and M2 (*Arg1*, *Mgl2* and *Mrc1*) related markers. In **a**-**c**, n = 5–6 mice per group. ^*^P < 0.05, ^**^P < 0.01 and ^***^P < 0.001 versus the Mye-CXCL14^+/+^_C group. **d-h** BMDMs were transfected with siRNAs for 24 h, and then treated with 200 μM 5MT for 24 h. BMDMs were polarized to M1 or M2 macrophages with LPS (60 ng/mL) or IL-4 (20 ng/mL). **d** M1 macrophages (F4/80^+^CD11c^+^) among BMDMs were detected by flow cytometry. **e**, **f** mRNA expression levels of genes indicative of macrophage M1 and M2 activation. **g** Protein expression levels of CXCL14, NOS2, Arg1, and phosphorylated and total P65. β-actin was used as the loading control. **h** mRNA expression levels of proinflammatory factors (*Tnf*, *Il1b* and *Il6*) in BMDMs. In **d**-**h**, n = 3; ^*^P < 0.05, ^**^P < 0.01 and ^***^P < 0.001 versus the C + siNC group; ^&^*P* < 0.05, ^&&^*P* < 0.01 and ^&&&^*P* < 0.001 versus the 5MT + siNC group. All data in bar plots are expressed as the mean ± S.E.M. ns, no significance

We analyzed the population of total macrophages (F4/80^+^), and the M1 (F4/80^+^CD11C^+^CD206^−^) and M2 (F4/80^+^CD11C^−^CD206^+^) macrophage subtypes isolated from liver tissues. Compared with Mye-CXCL14^+/+^ mice treated with vehicle, Mye-CXCL14^+/+^_5MT mice had fewer total macrophages (18.40% vs. 10.92%, *p* < 0.05) and M1 subtypes (M1/total macrophages: 43.18% vs. 29.72%, *p* < 0.05), but more M2 subtypes (M2/total macrophages: 5.43% vs. 8.01%, *p* < 0.05) (Fig. 5a). However, in the Mye-CXCL14^−/−^ mice, there was no significant change in the subpopulation of hepatic M1 or M2 macrophages after 5MT treatment. Similar results were observed when F4/80^+^NOS2^+^ macrophages were immunofluorescently labeled in the liver sections (Fig. 5b). Consistent with the changes in macrophage subpopulations, the 5MT-induced decrease in M1-related marker expression and increase in M2-related marker expression were attenuated in Mye-CXCL14^−/−^ mice (Fig. 5c).

Next, we assessed the role of 5MT in regulating macrophage polarization in vitro. Consistent with a previous study [32], the overexpression of *Cxcl14* inhibited the classical activation (M1) of BMDMs, and promoted alternative activation (M2) (Fig. S11a-c). Similarly, 5MT reduced the mRNA and protein expression of M1-related markers, and upregulated M2-related markers (Fig. S11b-d), subsequently inhibiting NF-κB activation and inflammatory factor expression (Fig. S11d, e). However, after *Cxcl14* was knocked down, the regulation of macrophage polarization mediated by 5MT was eliminated, as indicated by an increase in the number of M1 macrophages (Fig. 5d) and expression of M1-related markers, and a decrease in the expression of M2-related markers (Fig. 5e-g). Ultimately, the inhibition of NF-κB activity and inflammatory factor expression by 5MT was weakened after *Cxcl14* knockdown (Fig. 5g, h). In addition, the results observed in BMDMs were replicated in the macrophage-like cell line RAW264.7 (Fig. S12a-d). As mentioned above, these findings suggest that the 5MT-mediated inhibition of inflammation may depend, at least in part, on the upregulation of CXCL14 expression to suppress macrophage M1 polarization.

### 5MT upregulates macrophage Cxcl14 expression by promoting AHR-mediated transcriptional activation

**Fig. 6:**
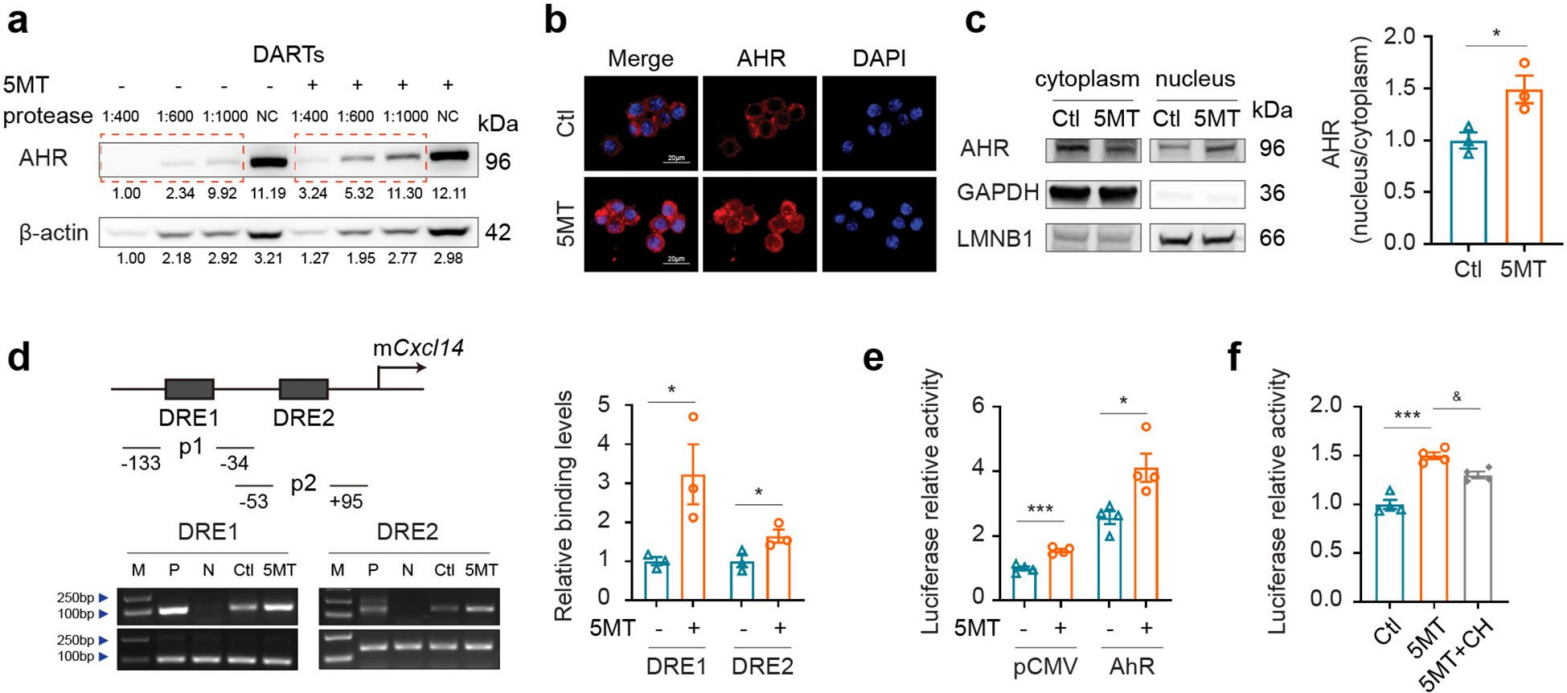
5MT upregulates macrophage CXCL14 expression by promoting AHR-mediated transcriptional activation. **a** The interaction of 5MT with the protein AHR was detected via DARTS assay. An aliquoted cell lysate was treated with 5MT or vehicle for 30 min at room temperature, and then incubated with the gradient diluted pronase for 15 min. **b-f** BMDMs were transfected with an AHR overexpression plasmid, and treated with 200 μM 5MT for 24 h and LPS (20 ng/mL) for 6 h. **b** IF staining of AHR in BMDMs. Scale bar, 20 μm. **c** Immunoblot analysis of AHR in the cytoplasm and nucleus of treated BMDMs. LaminB1 and GAPDH were used as the loading controls. **d** ChIP assays were performed to detect the potential binding sites (DREs) of AHR on the mouse Cxcl14 promoter. P, positive control (anti-RNA polymerase ll); N, negative control (anti-IgG). **e**, **f** A luciferase assay was used to assess the effects of 5MT on the transcriptional activation of the Cxcl14 promoter. **e** BMDMs were cotransfected with pCMV-AHR and the luciferase reporters CXCL14_promoter-firefly and TK promoter-Renilla, and then treated with 5MT in the presence of LPS. **f** Before 5MT treatment, the cells were pretreated with 10 μM CH223191 for 30 min. n = 3, all data in bar plots are expressed as the mean ± S.E.M. ^*^P < 0.05 and ^***^P < 0.001 versus the Ctl group; ^&^*P* < 0.05 versus the 5MT group

Considering that AHR mediates the role of 5MT, we examined and confirmed the binding of 5MT and AHR using the drug affinity responsive target stability (DARTS) assay, an analysis based on the decrease in protease susceptibility of a target protein after binding to its ligand [36]. The results shown in Fig. 6a indicate that 5MT increased the stability of AHR in cell lysates treated with gradient-diluted pronase. AHR is a ligand-activated transcription factor, and therefore, 5MT might promote *Cxcl14* expression through the transcriptional activation of AHR. As expected, after 5MT treatment, the translocation of AHR from the cytoplasm to the nucleus was clearly increased in BMDMs (Fig. 6b, c). An analysis using the Jaspar database revealed several putative dioxin response elements (DREs), which are DNA-binding sites for nuclear AHR, in the promoter sequence of the mouse *Cxcl14* gene. The upper panel in Fig. 6d shows DRE1 (−26 ~−31) and DRE2 (−77 ~ −82), whose relative scores (calculated by Jaspar) were > 0.9. Chromatin immunoprecipitation (ChIP) assays revealed that AHR was recruited to DREs in the *Cxcl14* gene promoter in BMDMs, and that this recruitment was enhanced by 5MT treatment (Fig. 6d). Moreover, the results of a dual-luciferase reporter gene assay revealed that 5MT strongly promoted the transcription of *Cxcl14* (Fig. 6e), whereas the inhibition of AHR activity with CH223191 suppressed the activation of the *Cxcl14* promoter (Fig. 6f). These findings indicate that 5MT upregulates the expression of *Cxcl14* by promoting AHR-mediated transcriptional activation.

### 5MT-CXCL14 inhibits classical macrophage activation by repressing glycolysis

**Fig. 7:**
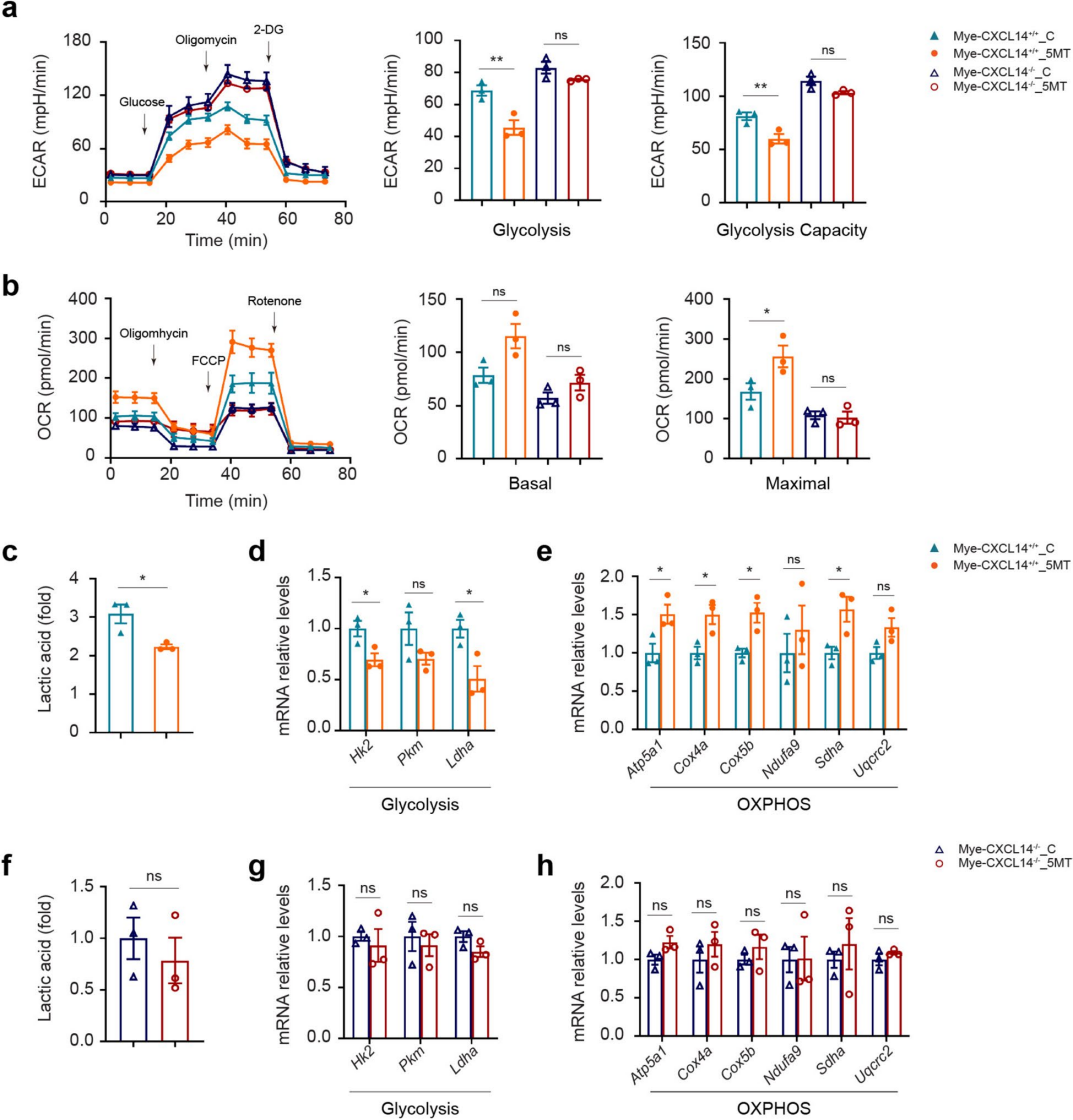
5MT-CXCL14 inhibits M1 polarization by repressing glycolysis in macrophages. BMDMs were isolated from Mye-CXCL14^+/+^ and Mye-CXCL14^−/−^ mice, and treated with 5MT (200 μM) in the presence of 20 ng/ml LPS. **a**, **b** Real-time rate changes in the ECAR (**a**) and OCR (**b**) were analyzed under basal conditions and then with sequential additions of various agonists and inhibitors at the indicated times. **c-e** Lactic acid production (**c**) and the mRNA expression levels of genes associated with glycolysis (**d**) and OXPHOS (**e**) were assessed in BMDMs isolated from Mye-CXCL14^+/+^ mice. **f–h** Lactic acid production (**f**) and the mRNA expression levels of genes associated with glycolysis (**g**) and OXPHOS (**h**) were assessed in BMDMs isolated from Mye-CXCL14^−/−^ mice. n = 3, all data in bar plots are expressed as the mean ± S.E.M. ^*^P < 0.05 and ^**^P < 0.01 versus the Mye-CXCL14^+/+^_C group; ns, no significance

Given that metabolic reprogramming is the main regulatory factor of macrophage polarization [37, 38], we monitored the macrophage extracellular acidification rate (ECAR) and oxygen consumption rate (OCR) using a respirometry and metabolomics instrument (Seahorse XF Mini extracellular flux analyzer). In BMDMs isolated from Mye-CXCL14^+/+^ mice, 5MT treatment resulted in a significant decrease in the ECAR and a notable increase in the OCR (Fig. 7a, b). However, in BMDMs isolated from Mye-CXCL14^−/−^ mice, the effects of 5MT on the cellular ECAR and OCR were weakened. BMDMs treated with 5MT exhibited a significant decrease in glycolytic capacity, manifesting as a decrease in the production of lactate and expression of glycolysis-related genes (*Hk2*, *Pkm* and *Ldha*, Fig. 7c, d). Moreover, the expression levels of oxidative phosphorylation (OXPHOS) subunits (*Atp5a1*, *Cox4a*, *Cox5b*, and *Sdha*) were obviously upregulated after 5MT treatment (Fig. 7e), indicating that 5MT induced a metabolic transition from glycolysis to oxidative phosphorylation in BMDMs. Specific knockout of *Cxcl14* inhibited the metabolic transition induced by 5MT in BMDMs (Fig. 7f-h). Taken together, these findings suggest that 5MT-CXCL14 inhibits glycolysis in BMDMs, leading to a phenotypic shift and functional changes in macrophages.

### Synergistic participation of macrophages and hepatocytes in the 5MT-mediated improvement of hepatic steatosis

Finally, to determine whether the inhibition of macrophage classical activation by 5MT-CXCL14 can improve insulin sensitivity in hepatocytes in vitro, we cocultured primary hepatocytes with conditioned medium (CM) from BMDMs isolated from Mye-CXCL14^+/+^ or Mye-CXCL14^−/−^ mice with or without 5MT pretreatment (Fig. S13a). After incubation, the insulin pathway was activated, and palmitic acid (PA)-induced lipid accumulation was significantly decreased in hepatocytes cocultured with CM from Mye-CXCL14^+/+^ -BMDMs pretreated with 5MT (Fig. S13b, c); moreover, the expression levels of gluconeogenic genes and glucose concentrations in the culture supernatant were reduced (Fig. S13d, e). However, CM from Mye-CXCL14^−/−^ -BMDMs pretreated with 5MT did not significantly affect insulin sensitivity, lipid deposition or gluconeogenesis in cocultured hepatocytes. Consistent with these findings after 5MT treatment, the presence of secreted proinflammatory factors (TNF-α, IL-1β and IL-6) in CM from Mye-CXCL14^+/+^ -BMDMs was obviously decreased, while the anti-inflammatory factor IL-10 was increased (Fig. S13f). In addition, compared with that in hepatocytes cocultured with CM_Mye-CXCL14^+/+^, AKT activity in hepatocytes cocultured with CM_Mye-CXCL14^−/−^ was inhibited to some extent, regardless of whether AHR activity was inhibited (Fig. S13g). These results confirm that 5MT improves hepatic insulin resistance in a macrophage CXCL14-dependent manner.

Given that previous studies have demonstrated that AHR is involved in regulating lipid metabolism in hepatocytes [15, 25, 39], we investigated the effects of 5MT on AHR activity and lipid metabolism in hepatocytes. As shown in Fig. S14a, 5MT upregulated the expression of AHR target genes in primary hepatocytes. Concurrently, the expression of fatty acid synthesis-related genes and PA-induced lipid deposition were significantly reduced (Fig. S14b-d). However, 5MT did not significantly affect gluconeogenesis in hepatocytes (Fig. S14e, f). After *Ahr* knockdown, the 5MT-mediated inhibition of fatty acid synthesis was attenuated, suggesting that 5MT may directly suppress fatty acid synthesis in hepatocytes via AHR. Taken together with the in vivo results, we speculate that the amelioration of hepatic steatosis by 5MT may result from a synergistic mechanism involving macrophages and hepatocytes.

In conclusion, we identified a novel role for the tryptophan derivative 5MT in improving hepatic insulin resistance and hepatic steatosis. Furthermore, we revealed that 5MT inhibits hepatic inflammation by activating the AHR/CXCL14 axis and regulating CXCL14-mediated macrophage polarization. By exploring the molecular pathway through which 5MT improves insulin resistance, our findings provide a theoretical basis for new treatments for metabolic diseases, such as obesity and diabetes.

## Discussion

Accumulating evidence indicates that tryptophan metabolism plays important roles in regulating the inflammatory response, insulin sensitivity and metabolic homeostasis [40, 41]. Metabolic disorders are associated with a reduced capacity of the gut microbiota to metabolize tryptophan into indole derivatives [42]. To the best of our knowledge, this study is the first to report that the tryptophan metabolite 5MT acts as a ligand that activates AHR to ameliorate hepatic insulin resistance in HFD and db/db mice.

In this study, serum levels of 5MT were reduced in obese and diabetic mice, but recovered following treatment with the antidiabetic agent DPP-4i. Consistent with our findings, in a previous longitudinal cohort study, logistic regression analysis revealed that 5MT may serve as a predictor of the restoration from prediabetes to normal glucose regulation, when combined with four other metabolites [21]. Another study revealed that 5MT levels were significantly reduced at the hepatic cirrhosis stage, and were negatively correlated with liver fibrosis markers [43]. The findings from those studies indicate that 5MT is closely associated with metabolic disorders, and our results validate this relationship. However, the administration of tryptophan to high-fat and high-fructose diet-fed mice has been shown to exacerbate hepatic steatosis [44]. 5MT is a derivative of tryptophan, which can be catabolized by a large number of gut bacteria (e.g., *Bacteroides*) to produce various indole derivatives (e.g., indole acetate, indole lactate and indole propionate) [7]. Upon entering the circulatory system, indole derivatives exert anti-inflammatory effects and improve metabolism at extragastrointestinal sites, such as the liver. For instance, compared with those in controls, the fecal levels of indole-3-acetic acid (IAA) and indole-3-propionic acid (IPA) in HFD mice and patients with hepatic steatosis are lower. IAA and IPA supplementation has been shown to ameliorate hepatic steatosis and inflammation induced by a Western diet or fatty acids [15, 45]. Our research on 5MT further highlights the critical effect of tryptophan metabolites on metabolic homeostasis, potentially providing a novel therapeutic strategy for metabolic disorders.

Indole derivatives have been identified as ligands for AHR to exert their biological effects [42]. Upon ligand binding, cytoplasmic AHR undergoes nuclear translocation, which facilitates its heterodimerization with AHR nuclear translocator (ARNT/HIF1β), forming a competent transcription factor that drives the expression of AHR target genes, such as *Cyp1a1* and *Il22* [24, 46]. By integrating transcriptome sequencing with a molecular mechanism investigation, our study revealed that 5MT interacts with AHR and promotes the transcription of *Cxcl14* in macrophages, consequently repressing M1 polarization and the inflammatory response. Chronic inflammation is present at the onset of glucose intolerance and insulin resistance [47]. Inflammatory factors can inhibit the binding of insulin receptor substrate (IRS-1) to the insulin receptor, and reduce the tyrosine phosphorylation of IRS-1, thereby leading to the sustained development of insulin resistance [48]. The pathological retention or prolonged activation of macrophages in the liver is a canonically important aspect of inflammation, that in turn contributes to insulin resistance [33, 34]. CXCL14, also known as breast and kidney-expressed chemokine (BRAK), has been reported to promote noninflammatory alternative macrophage activation and suppress M1 macrophage infiltration [30, 32]. Additionally, the serum levels of CXCL14 have been shown to be significantly lower in obese T2DM subjects than in healthy controls, and to be negatively associated with insulinemia and HOMA-IR [49]. These findings demonstrate that CXCL14 is involved in the pathogenesis of inflammation and metabolic disorders. On the basis of our results, 5MT may function primarily in improving insulin resistance by at least partially inhibiting inflammation through AHR/CXCL14.

Mechanistically, immunometabolism studies have shown that alterations in the metabolic profile of macrophages shape their activation status and function [50]. The inhibition of glycolysis can stimulate peritoneal macrophages to switch from a proinflammatory phenotype to a reparative phenotype [51]. Notably, CXCL14 has been reported to be involved in regulating glycolysis [30]. Therefore, from the perspective of metabolic reprogramming, we preliminarily explored the mechanism through which the 5MT/CXCL14 axis regulates macrophage polarization. These data indicate that 5MT inhibited glycolysis in BMDMs, as evidenced by reduced ECAR and lactate production following LPS stimulation. However, the downstream molecular mechanisms of CXCL14 in metabolic remodeling need to be explored further.

Upon the coculture of hepatocytes with CM from BMDMs, we confirmed that 5MT activated the insulin signaling pathway and reduced fat deposition by inhibiting the expression of macrophage-derived proinflammatory factors, a process that is dependent on macrophage CXCL14. These findings provide a strong basis for determining the pivotal role of macrophage CXCL14 in the ability of 5MT to ameliorate insulin resistance and promote metabolic homeostasis. Moreover, we found that 5MT has a direct regulatory effect on fatty acid synthesis in hepatocytes through AHR. These results suggest that the 5MT-induced improvement in hepatic steatosis in obese and diabetic mice may be attributed to the synergistic effect of macrophages and hepatocytes.

The present study has several limitations that need to be noted. First, no clinical samples from patients with diabetes or obesity were used to validate the relationship between 5MT and insulin resistance. Second, the effects of 5MT on other metabolic organs were not thoroughly investigated, and only preliminary assessments were made regarding changes in the expression of fatty acid metabolism-related markers in adipose tissues. 5MT was detected in liver, muscle and WAT, although its levels varied among tissues. However, whether 5MT exerts distinct effects across different metabolic organs, and whether such functional heterogeneity is associated with differential 5MT accumulation remain unclear. These issues warrant further investigation in future studies.

In conclusion, we identified a novel role for the tryptophan derivative 5MT in improving hepatic inflammation and insulin resistance. Furthermore, this study revealed that 5MT regulates CXCL14-mediated macrophage polarization by activating AHR, thereby suppressing the hepatic inflammatory response. By exploring the molecular pathway through which 5MT improves insulin resistance, our findings provide a theoretical basis for new treatments for metabolic diseases, such as obesity and diabetes.

## Materials and methods

### Mice and treatment

Male C57BL/6 J, db/m, db/db (C57BLKS/JGpt background), CXCL14-flox (CXCL14^fl/fl^, C57BL/6 J background), and Lyz2-iCre (C57BL/6 J background; the expression of cyclization recombinase (Cre) is under the control of the lysosome M promoter) mice were purchased from the GemPharmatech Co., Ltd, China. CXCL14^fl/fl^ mice were bred with Lyz2-iCre mice to generate myeloid cell-specific CXCL14 knockout (Mye-CXCL14^–/–^) mice and littermate controls (Mye-Cxcl14^+/+^). Male C57BL/6 J mice (5–6 weeks old) were fed a normal chow diet (NCD) or a high-fat diet (HFD, 60% fat, 20% protein, 20% carbohydrate (kcal/100 g)) for 14 weeks. Male db/db mice (7–8 weeks) were fed an NCD before and during treatment. All the mice were housed at 25 °C under a 12 h light/dark cycle, and had free access to water and food. **Study I**: HFD and db/db mice were divided into different groups based on matched weights and fasting blood glucose (FBG) levels. The DPP-4i treatment groups were treated with 4 g/kg sitagliptin (Sit, Merck Sharp & Dohme, USA) or vehicle mixed with an NCD or HFD for 4 weeks. **Study II**: C57BL/6 J mice aged 8–9 weeks were orally administered 5MT at dosages of 5, 10, and 30 mg/kg per day for 6 weeks, after which liver and kidney function-related indicators were tested. **Study III**: The NCD, HFD and db/db (9–10 weeks) mice were divided into different groups based on matched weight and FBG levels, and treated with 5MT (orally, 30 mg/kg, Sigma-Aldrich) or vehicle for the last 6 weeks during the HFD feeding period. **Study IV**: To inhibit AHR activity, NCD (9–10 weeks) mice were administered CH-223191 (i.p., 10 mg/kg) for 10 days. HFD mice were administered CH-223191 (10 mg/kg) for the last 10 days during the 5MT treatment period. **Study V**: Male Mye-CXCL14^–/–^ and Mye-CXCL14^+/+^ mice, aged 5–6 weeks, were fed a HFD for 8 weeks, and then treated with 5MT or vehicle for 6 weeks as described in Study II. The details of the diets and reagents used are listed in Table S2.

### Primary cell isolation, culture and treatment

Mouse bone marrow-derived macrophages (BMDMs) were isolated from bone marrow cells prepared from the tibias and femurs of mice aged 8–10 weeks. BMDMs were cultured in macrophage medium MaM (ScienCell Research Laboratories) supplemented with 1% macrophage growth supplement, 5% fetal bovine serum (FBS), 1% penicillin/streptomycin and recombinant murine M-CSF (20 ng/ml, SinoBiological) to induce differentiation as previously described [52]. The purity and differentiation of BMDMs were determined by flow cytometry analysis (FACS) of F4/80 and CD11b expression. After differentiation, the BMDMs were treated with gradient dilutions of 5MT (10 ~ 2000 μM) for 24 h, and cell activity was assessed using a CCK-8 assay kit (Selleck). RAW 264.7 macrophages were cultured in DMEM supplemented with 10% FBS and 1% penicillin/streptomycin. BMDMs and RAW264.7 macrophages were serum-starved for 6 h, and then polarized to M1 or M2 macrophages with LPS (60 ng/mL, MedChemExpress) or IL-4 (20 ng/mL, SinoBiological) for 18 h [32]; the cells were then used for polarization and inflammation assays after 5MT treatment.

Mouse primary hepatocytes were isolated from the livers of male C57BL/6 J mice (7–8 weeks old) through liver perfusion. Briefly, after the mice were anesthetized, hepatocytes were isolated via injection of 0.35 mg/mL type IV collagenase (Sigma-Aldrich) through the inferior vena cava and separated by 40% Percoll gradient (Solarbio) centrifugation (50 × g) [53]. Trypan blue dye was used to detect cell activity. Primary hepatocytes were subsequently cultured in low-glucose (1 g/L) DMDM (Gibco) supplemented with 10% FBS (Gibco), 100 nM dexamethasone (MedChemExpress), 0.5 μg/mL insulin (MedChemExpress) and 1% penicillin–streptomycin (HyClone). Cell culture plates were precoated with 12 μg/mL collagen type I (Solarbio). Hepatocytes were identified by cell morphology and IF staining with an anti-Albumin antibody (Proteintech, 1:100).

Liver macrophages were enriched and isolated in accordance with a modified protocol based on a previous study [54]. Nonparenchymal cells (NPCs) were suspended in HBSS and layered onto a two-layer 30%−70% Percoll gradient (Solarbio) in a 50 mL conical centrifuge tube, 1800 g at 4 °C for 15 min. Liver macrophages in the middle layer were collected and allowed to attach to cell culture plates in RPMI-1640 medium (Servicebio) supplemented with 10% FBS for 30 min at 37 °C. Nonadherent cells were removed by replacing the culture medium. The purity of the adherent macrophages was determined by IF staining with an anti-F4/80 antibody. Detailed information on the reagents used for isolating primary cells is presented in Table S2.

For coculture, BMDMs isolated from Mye-Cxcl14^+/+^ or Mye-CXCL14^–/–^ mice were pretreated with 5MT for 24 h, followed by LPS stimulation. The culture supernatants were subsequently collected as conditioned medium (CM). To assess lipid metabolism and insulin pathways, primary hepatocytes were cocultured with CM for 24 h, and then stimulated with PA (0.3 mM, 12 h) and insulin (100 nM, 30 min). To assess gluconeogenesis, cocultured hepatocytes were incubated with lactate (10 mM) and propionate (1 mM) under glucose- and serum-free conditions 8 h prior to harvesting. Glucose concentrations in the culture supernatant were measured using a commercial detection kit (Table S2).

### Statistical analysis

Quantitative data are presented as the mean ± S.E.M. Student's t test was used to compare data between two groups. To compare multiple groups, differences were analyzed by one-way ANOVA with FDR correction or Tukey's test. A *P* value < 0.05 was defined as statistically significant.

## Supplementary Information

Supplementary Material 1.

## Data Availability

The RNA-seq data of mouse livers produced in this study have been deposited in the Sequence Read Archive database under accession number: SRR30970541, SRR30970542, SRR30970543 and SRR30970544, and are available at the following URL: https://www.ncbi.nlm.nih.gov/sra/?term = SRR30970541.
