# Supplementary material for: 5-methoxytryptamine improves hepatic inflammation and insulin resistance in a macrophage C-X-C motif chemokine ligand 14 dependent manner
Source: Mol Biomed. 2026 Jun 28;7:101. doi: 10.1186/s43556-026-00507-3 (PMC13310876; doi:10.1186/s43556-026-00507-3)
Supplement: Supplementary file 1 — Supplementary Material 1. [file 43556_2026_507_MOESM1_ESM.pdf]

5-methoxytryptamine improves hepatic inflammation and insulin resistance in a macrophage C-X-C motif chemokine ligand 14 dependent manner

Xiaoyu Liao[1,†,*], Yuxi Xiao[1,†], Bingyao Liu[1,2,†], Yuan Dong[1,†], Hang Yang[1,3,†], Linlin Zhang[1], Dong Li[4], Peiye Sun[1], Yixiang Feng[1], Ying Yang[5], Liqun Zhang[5], Qingwu Yang[6], Qiang Tong[1], Hongting Zheng[1,*]

Author Affiliations:

[1] Department of Endocrinology, Metabolic and Chronic Disease Science Innovation Center, Translational Research of Diabetes Key Laboratory of Chongqing, the Second Affiliated Hospital of Army Medical University, Chongqing, 400037, China.

[2] Department of General Medicine, General Hospital of Western Theater Command, Chengdu, Sichuan, 610083, China.

[3] Department of Endocrinology, General Hospital of Western Theater Command, Chengdu, Sichuan, 610083, China.

[4] Department of Endocrinology, Chongqing Hospital of Traditional Chinese Medicine, Chongqing, 400021, China.

[5] Department of Clinical Laboratory Medicine, the Second Affiliated Hospital of Army Medical University, Chongqing, 400037, China.

[6] Department of Neurology, the Second Affiliated Hospital of Army Medical University, Chongqing, 400037, China.

[†] These authors contributed equally to this work.

* Corresponding authors.

Corresponding authors:

Hongting Zheng, M.D., Ph.D., E-mail: fnf7703@hotmail.com; zhenghongting@tmmu.edu.cn.

Department of Endocrinology, Metabolic and Chronic Disease Science Innovation Center, Translational Research of Diabetes Key Laboratory of Chongqing, the Second Affiliated Hospital of Army Medical University, Chongqing, 400037, China.

Phone: +8602368755709, Fax: +8602368755707.

Xiaoyu Liao, Ph.D., E-mail: liaoxiaoyu2010@163.com; liaoxiaoyu@tmmu.edu.cn.
Department of Endocrinology, Metabolic and Chronic Disease Science Innovation Center, Translational Research of Diabetes Key Laboratory of Chongqing, the Second Affiliated Hospital of Army Medical University, Chongqing, 400037, China.
Phone: +8602368774669.

Supplementary files

Supplementary Materials and Methods:

Supplementary Figures:

Fig. S1. Serum 5MT levels are lower in HFD and db/db mice

Fig. S2. 5MT has no side effects on liver or kidney structure or function in C57BL/6J mice

Fig. S3. 5MT inhibits fatty acid synthesis in the iWAT of HFD and db/db mice

Fig. S4. Inhibiting AhR abolishes the upregulation of Cyp1a1 and Ahrr expression induced by 5MT

Fig. S5. CH223191 has no significant effect on hepatic insulin resistance and lipid metabolism in NCD mice

Fig. S6. 5MT mainly affects the hepatic inflammatory response in HFD mice

Fig. S7. The expression levels of CXCL14 in different primary cells

Fig. S8. Identification of myeloid-specific CXCL14 knockout mice

Fig. S9. 5MT improves hepatic steatosis in a macrophage CXCL14-dependent manner

Fig. S10. 5MT reduces the number of M1 macrophages in the liver of HFD mice

Fig. S11. 5MT regulates macrophage polarization

Fig. S12. 5MT regulates macrophage polarization through CXCL14 in RAW264.7 cells

Fig. S13. 5MT activates the insulin pathway in hepatocytes in a macrophage CXCL14-dependent manner

Fig. S14. 5MT inhibits fatty acid synthesis in hepatocytes via AHR

Supplementary Tables:

Table S1. The significantly changed genes after 5MT treatment

Table S2. List of reagents and kits

Table S3. List of antibodies

Table S4. Sequences of siRNA and primers (for mouse)

Supplementary Materials and Methods

Measurement of serum 5MT concentration by LC-MS/MS

Metabolite extraction: A 120 μL aliquot of each serum sample was added to 480 μL of extraction solvent (acetonitrile: methanol=1:1, containing an isotopically- labeled internal standard, precooled at -40°C), vortexed and sonicated for 15 min in an ice-water bath. Then the samples were incubated for 1 h at -40°C, and centrifuged at 12000 rpm for 15 min at 4°C. The supernatant was collected and dried by spinning. The residue was reconstituted with 100 μL of precooled extraction solvent (acetonitrile: methanol: water = 2:2:1), vortexed and sonicated for 5 min in an ice-water bath; the reconstituted samples were centrifuged at 12000 rpm for 15 min at 4°C. The supernatant was subjected to LC-MS/MS analysis (Shanghai Biotree Biotech Co., Ltd.). UHPLC separation was carried out using an Agilent 1290 Infinity II series UHPLC System (Agilent Technologies), equipped with a Waters ACQUITY UPLC HSS T3 (100 × 2.1 mm, 1.8 μm, Waters). An Agilent 6460 triple quadrupole mass spectrometer (Agilent Technologies) equipped with an AJS electrospray ionization (AJS-ESI) interface was used for assay development. MRM data acquisition and analysis were performed using Agilent MassHunter Work Station Software (B.08.00, Agilent Technologies).

Glucose tolerance test (GTT) and insulin tolerance test (ITT)

GTTs and ITTs were conducted during the sixth week of 5MT treatment. The mice were fasted overnight (12-14 hours) for the GTT or fasted 4-6 hours for the ITT as described in previous studies [1], and then injected intraperitoneally with a solution of glucose at 1 g/kg body weight, or with insulin (MedChemExpress) at 0.75 U/kg body weight for the HFD and 1 U/kg body weight for the db/db mice. Tail blood glucose levels were measured at different time points after injection using an Accu-Check glucometer (Roche, Basel, Switzerland). Fasting insulin levels were measured using a Rat/Mouse Insulin ELISA kit (Millipore, Bedford, MA, USA). The homeostasis model assessment of insulin resistance (HOMA-IR) was calculated as previously described: HOMA-IR = (fasting glucose (mmol/L) × fasting insulin (mU/L)) / 22.5 [2].

Hematoxylin and Eosin (H&E) and periodic acid-Schiff (PAS) staining

H&E and PAS staining were performed as previously described [3]. All images were obtained using an Olympus fluorescence microscope. Details regarding the commercial assay kits are provided in Table S2.

Oil red O staining

Lipid droplet accumulation in livers and hepatocytes was assessed using Oil red O staining. Frozen liver sections were prepared in Tissue-Tek Opti-mum Cutting Temperature (OCT) compound (SAKURA, USA), and hepatocytes were fixed in formaldehyde solution. Oil red O (Sigma-Aldrich) was prepared as a 0.5% (m/v) stock solution, and diluted with deionized water at a 3:2 ratio prior to staining. Staining was performed as previously described [4]. Details regarding the reagents used are provided in Table S2.

Immunofluorescence (IF) staining

IF staining was performed as described previously [5]. The following primary antibodies were used: anti-F4/80 (CST, 1:100), anti-NOS2 (Proteintech, 1:50), anti-AHR (Proteintech, 1:100), anti-Albumin (Proteintech, 1:100) and anti-α-SMA (CST, 1:100).

Measurement of lipid contents in the liver, serum and hepatocytes

Triglyceride (TG), total cholesterol (TC), and nonesterified fatty acids (NEFA) in liver tissues, serum and hepatocytes were measured using the corresponding commercial kits in accordance with the manufacturer’s instructions (Nanjing Jiancheng Bioengineering Institute).

Measurement of liver and kidney function indices and inflammatory factors

The serum levels of liver function indices, including alanine aminotransferase (ALT), aspartate aminotransferase (AST) and alkaline phosphatase (ALP) in treated and control mice were measured using assay kits purchased from Nanjing Jiancheng Bioengineering Institute. Serum creatinine, urinary albumin, and creatinine levels were measured using commercial testing kits (Nanjing Jiancheng Bioengineering Research Institute) in accordance with the manufacturer's protocol. The levels of inflammatory factors including TNFα, IL-1β, IL-6 and IL-10 in the serum of treated mice or in the cell culture medium were measured with commercial kits in accordance with the manufacturer’s instructions (Multi Sciences).

Flow cytometry analysis of macrophages

To analyze the population of the M1 and M2 macrophage subtypes, liver macrophages and treated BMDMs were preincubated with an anti-CD16/CD32 antibody (BioLegend) for 10 min at 4°C to block nonspecific binding, and incubated with Fixable Viability Dye (BioLegend) for 15 min in the dark. Then, the cells were stained with anti-F4/80, anti-CD11c and anti-CD206 antibodies. All stained cells were examined on a FACS Caliber flow cytometer (Beckman), and

the data were analyzed using FlowJo software (FlowJo, LLC, Ashland, OR, USA). The antibodies used are listed in Table S3.

Drug affinity responsive target stability (DARTS) assay

The DARTS assay was performed as previously described [6]. BMDMs were seeded in 10 cm plates, and transfected with pCMV3-AHR for 24 hours. The treated cells were lysed with 600 μL of lysis buffer, followed by the addition of 66 μL 10× TNC buffer. The cell lysate was divided into 99 μL aliquots, to which 1μL of 100× 5MT (20 mM) or vehicle was added, followed by incubation at room temperature for 30 min. Then, the mixture was aliquoted into 5 parts, and each 20 μL sample was incubated with 2 μL of gradient-diluted pronase (1:400, 1:600, and 1:1000) for 15 min, after which proteolysis was terminated by incubation on ice for 10 min. Finally, the prepared samples were analyzed by immunoblotting.

The lysis buffer (1.0 mL) consisted of 50 μL of 20x protease inhibitors (Roche), 50 μL of 1M sodium fluoride, 100 μL of 100 mM β-glycerophosphate, 100 μL of 50 mM sodium pyrophosphate, 10 μL of 200 mM sodium orthovanadate, and 690 μL of M-PER reagent (Thermo Scientific). The 10× TNC buffer (1mL) consisted of 500 μL of 1M Tris-HCL (pH = 8.0), 100 μL of 5M NaCl, 100 μL of 1M CaCl2, and 300 μL of ultrapure water. After preparation, aliquots were stored at -20°C. The protease inhibitor cocktail (20×) contained one tablet of protease inhibitor (Roche) dissolved in 525 μL of ultrapure water. Pronase was dissolved at 10mg/mL and stored at -20°C. Detailed information regarding the above reagents is provided in Table S2.

RNA sequencing (RNA-Seq)

Total RNA was extracted from liver tissues from 5MT or vehicle treated HFD mice. RNA integrity and concentration were assessed using an Agilent 2100 Bioanalyzer (Agilent Technologies). RNA-Seq was performed by Majorbio Bio-pharm Technology Co., Ltd. (Shanghai, China) using an Illumina HiSeq xten/NovaSeq 6000 sequencer. Finally, the sequencing data analysis was performed using the free online platform of Majorbio Cloud Platform (www.majorbio.com).

Plasmid construction, siRNA and plasmid transfection

Overexpression vectors were constructed by cloning the coding sequences of mouse AhR and mouse CXCL14 into the pCMV3 vector (pCMV3-AhR and pCMV3-CXCL14). The constructed vectors were verified by Sanger sequencing. siRNAs of CXCL14 were synthesized by Sangon Biotech (Shanghai, China). Plasmids and siRNAs were transfected with

Lipofectamine 3000 (Invitrogen) or Lipofectamine RNAiMAX (Invitrogen) respectively, in accordance with the manufacturer's instructions. The siRNA sequences are listed in Table S4.

Oxygen consumption rate (OCR), extracellular acidification rate (ECAR) and lactic acid assay

OCR was assessed using a Seahorse XF HS Mini analyzer (Agilent Technologies) in accordance with our previous description [7]. Briefly, BMDMs were seeded in Seahorse XF cell culture miniplates (7-8 × 10^4 cells in 100μL of growth medium per well), and treated with 5MT (200 μM) in the presence of 20 ng/ml LPS. Before measurement, the cells were washed and cultured (at 37°C in a non-CO2 incubator) in test medium (DMEM supplemented with glucose, pyruvate and glutamine, Agilent Technologies) for 1 hour. The following compounds were subsequently added: 2.5 μM oligomycin A, 2.5 μM FCCP, and 2.5 μM rotenone. The cell OCR was automatically recorded by the sensor cartridge and calculated using the Seahorse XF Mini software. To determine the ECAR, cells were washed and cultured in DMEM supplemented with glutamine before measurement. Then, the following compounds were added: 10 mM glucose, 1.5 μM oligomycin, and 50 mM 2-deoxy-D-glucose (MCE). The cells were then analyzed using a glycolysis stress test. To determine lactic acid production, BMDMs were treated with 5MT in the presence of LPS, and were subsequently collected at the indicated time points. Lactic acid production was measured using a lactic acid assay kit (Nanjing Jiancheng Bioengineering Institute). Detailed information regarding the above reagents is provided in Table S2.

Chromatin immunoprecipitation (ChIP)

BMDMs were transfected with the overexpression vector pCMV3-AhR and treated with 200 μM 5MT or vehicle. The cells were fixed with 1% formaldehyde to crosslink proteins and DNA, and then lysed and sonicated to shear DNA to 200-1000bp. The sonicated protein/DNA was incubated with anti-AhR antibody and protein A/G magnetic beads (Millipore) overnight at 4°C with rotation. Then, the protein A/G bead-antibody/chromatin complex was reversed and purified to remove the chromatin proteins. Finally, the purified free DNA was amplified by PCR and quantified using ImageJ software. The primers used for PCR are listed in Table S4.

Dual-luciferase reporter gene assay

A dual-luciferase reporter gene assay was performed as described previously [3, 8]. BMDMs were cotransfected with the overexpression plasmid pCMV3-AhR, and luciferase reporter plasmids CXCL14_promoter-firefly and TK promoter-Renilla (internal control). After twenty-

four hours later, the cells were treated with 5MT in the presence of LPS. Then, Firefly and Renilla luciferase activities were subsequently determined using the Dual-luciferase reporter gene assay system (Promega).

Immunoblot assay

Tissues or cells were lysed with lysis buffer containing protease inhibitor cocktail (MedChemExpress) and phosphatase inhibitor cocktail (MedChemExpress). After determining the protein concentration, cell lysates were subjected to immunoblot analysis using the indicated primary antibodies and their corresponding HRP-conjugated secondary antibodies (Table S3). Blots were developed with ECL (Thermo Scientific Pierce) and imaged using a fusion FX5s system (Vilber Lourmat).

Quantitative Real-Time PCR (qRT-PCR)

Total RNA was isolated from tissues or treated cells using RNAiso Plus (Takara) in accordance with the manufacturer's instructions. RNA concentrations were assessed using a Nanodrop 2000 (Thermo Fisher Scientific). RNA (1000 ng) was reversed transcribed into cDNA using a PrimeScript RT Reagent Kit with gDNA Eraser (Takara). TB Green Premix (Takara) was used to quantify the amplified PCR products. *Gapdh* or *Acta2* was used as the reference gene for normalization. The primer sequences are presented in Table S4.

Reference

1. Sun N, Shen C, Zhang L, Wu X, Yu Y, Yang X *et al.* Hepatic kruppel-like factor 16 (klf16) targets pparalpha to improve steatohepatitis and insulin resistance. *Gut*. 2021;**70**:2183-95 https://doi.org/10.1136/gutjnl-2020-321774
2. Cai D, Yuan M, Frantz DF, Melendez PA, Hansen L, Lee J *et al.* Local and systemic insulin resistance resulting from hepatic activation of ikk-beta and nf-kappab. *Nature medicine*. 2005;**11**:183-90 https://doi.org/10.1038/nm1166
3. Liu B, Zhang L, Yang H, Chen X, Zheng H, Liao X. Sik2 protects against renal tubular injury and the progression of diabetic kidney disease. *Translational research : the journal of laboratory and clinical medicine*. 2023;**253**:16-30 https://doi.org/10.1016/j.trsl.2022.08.012
4. Liao X, Song L, Zhang L, Wang H, Tong Q, Xu J *et al.* Lamp3 regulates hepatic lipid metabolism through activating pi3k/akt pathway. *Molecular and cellular endocrinology*. 2018;**470**:160-67 https://doi.org/10.1016/j.mce.2017.10.010
5. Wang H, Liu X, Long M, Huang Y, Zhang L, Zhang R *et al.* Nrf2 activation by antioxidant antidiabetic agents accelerates tumor metastasis. *Science translational medicine*. 2016;**8**:334ra51 https://doi.org/10.1126/scitranslmed.aad6095

6. Lomenick B, Hao R, Jonai N, Chin RM, Aghajan M, Warburton S *et al.* Target identification using drug affinity responsive target stability (darts). *Proceedings of the National Academy of Sciences of the United States of America*. 2009;**106**:21984-9 https://doi.org/10.1073/pnas.0910040106
7. Liu X, Qu H, Zheng Y, Liao Q, Zhang L, Liao X *et al.* Mitochondrial glycerol 3-phosphate dehydrogenase promotes skeletal muscle regeneration. *EMBO molecular medicine*. 2018;**10**:e9390 https://doi.org/10.15252/emmm.201809390
8. Qin HD, Liao XY, Chen YB, Huang SY, Xue WQ, Li FF *et al.* Genomic characterization of esophageal squamous cell carcinoma reveals critical genes underlying tumorigenesis and poor prognosis. *American journal of human genetics*. 2016;**98**:709-27 https://doi.org/10.1016/j.ajhg.2016.02.021

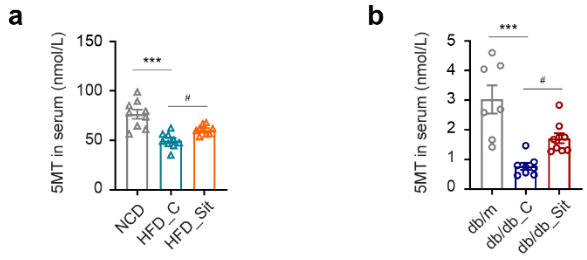

Fig. S1. Serum 5MT levels are lower in HFD and db/db mice.

(**a**) The concentrations of 5MT in the serum of NCD mice, and HFD mice treated with 4 g/kg sitagliptin (Sit) or vehicle for 4 weeks. (**b**) The concentrations of 5MT in the serum of db/m mice and db/db mice treated with 4 g/kg Sit or vehicle for 4 weeks. n=7-9 mice per group; all data in bar plots are expressed as the mean ± S.E.M. $^{***}P < 0.001$ and $^{\#}P < 0.05$.

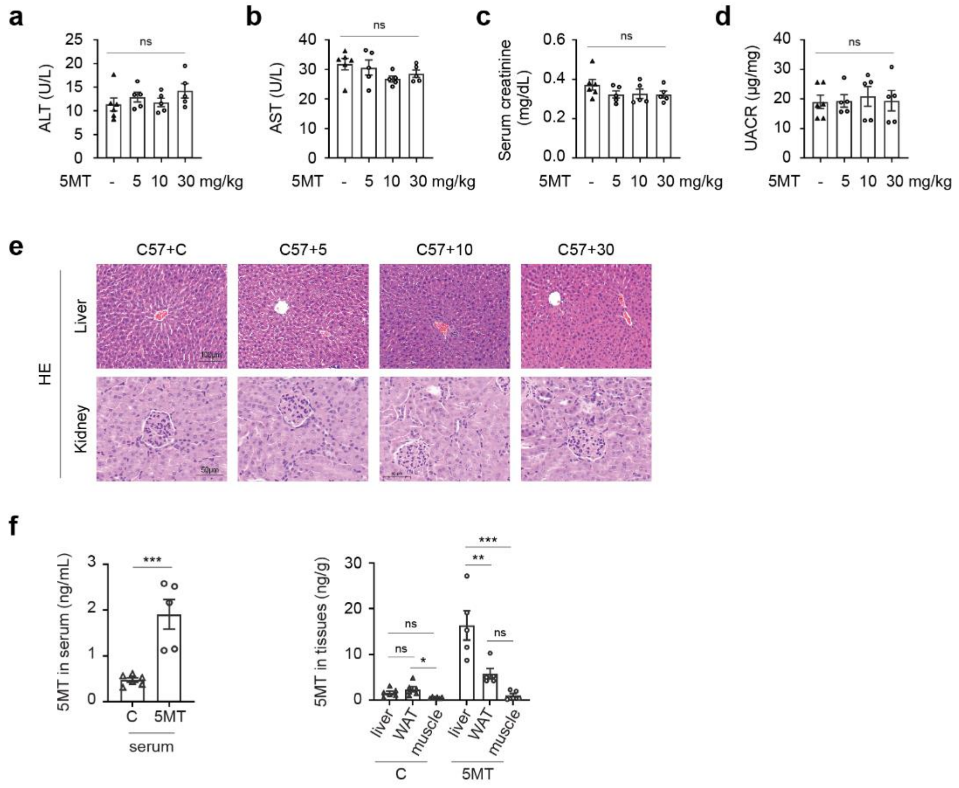

Fig. S2. 5MT has no side effects on liver or kidney structure or function in C57BL/6J mice.

C57BL/6J mice aged 8-9 weeks were orally administered 5MT at dosages of 5, 10, and 30 mg/kg per day for 6 weeks. (**a**, **b**) Serum ALT and AST levels in each group. (**c-d**) The serum creatinine level and urine albumin-to-creatinine ratio (UACR) in each group. (**e**) Histological images of liver and kidney sections from the indicated mice stained with H&E. (**f**) 5MT levels in the serum and tissues of C57BL/6J mice treated with vehicle or 30 mg/kg 5MT were measured by LC-MS. Scale bar in liver, 100 μm; scale bar in kidney, 50 μm. n=5-6 mice per group; all data in bar plots are expressed as the mean ± S.E.M. *P < 0.05 and ***P < 0.001; ns, no significance.

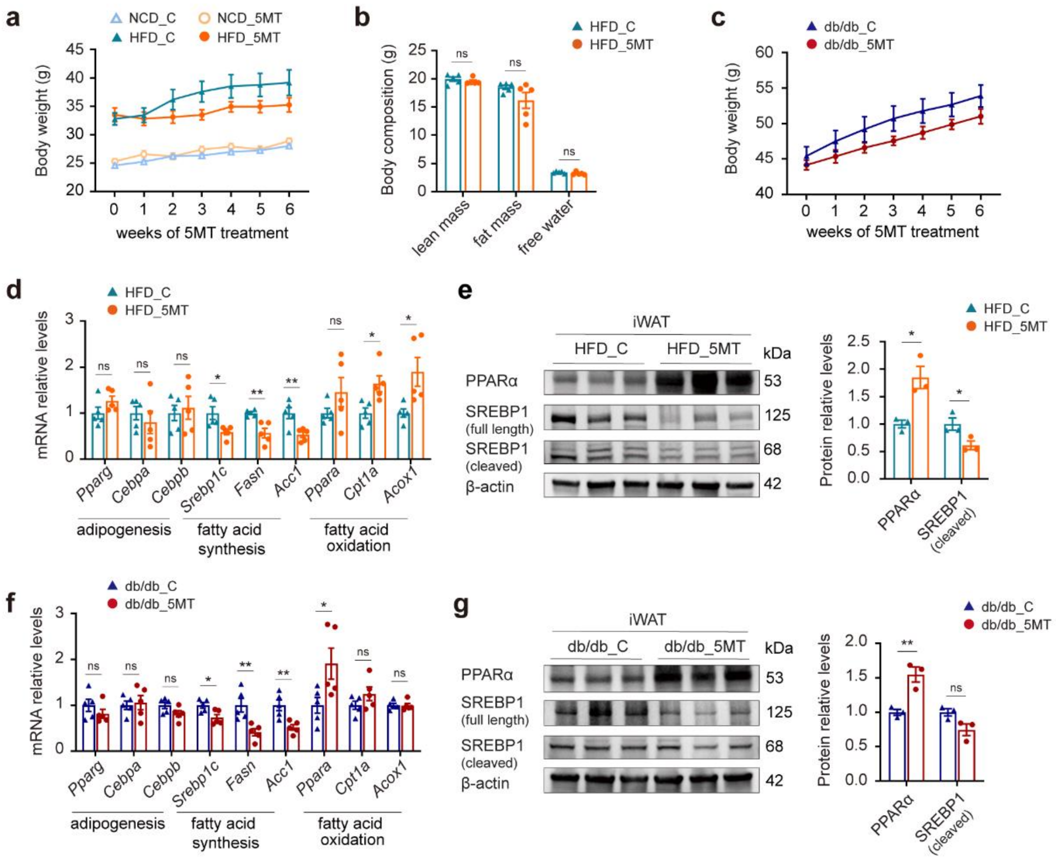

Fig. S3. 5MT inhibits fatty acid synthesis in the iWAT of HFD and db/db mice.

(**a**) The body weights of NCD and HFD mice were measured every week during the 5MT treatment period. (**b**) Fat mass, lean mass and free water content in HFD mice treated with or without 5MT. (**c**) The body weight of db/db mice was measured every week during 5MT treatment period. (**d, e**) mRNA and protein expression levels of markers regulating adipogenesis, fatty acid synthesis, and fatty acid oxidation in the iWAT of treated HFD mice. β-actin was used as the loading control. (**f**, **g**) mRNA and protein expression levels of markers regulating lipid metabolism in the iWAT of treated db/db mice. β-actin was used as the loading control. n=4-5 mice per group; all data in bar plots are expressed as the mean ± S.E.M. *$P < 0.05$ and **$P < 0.01$, ns, no significance.

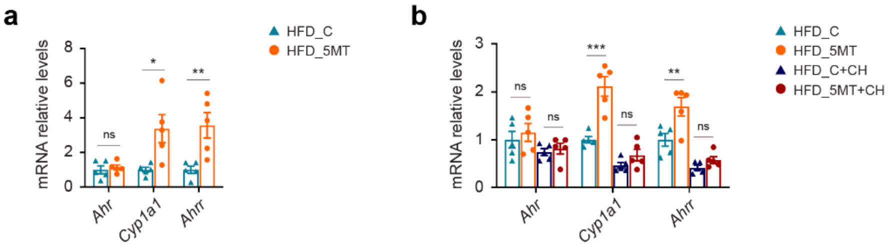

Fig. S4. Inhibiting AhR abolishes the upregulation of Cyp1a1 and Ahrr expression induced by 5MT.

(**a**) mRNA expression levels of AhR and AhR target genes, including Cyp1a1 and Ahrr, in the liver of HFD_C and HFD_5MT mice. (**b**) mRNA expression levels of AhR and AhR target genes in the livers of HFD mice treated with 5MT and/or CH223191. n=5 mice per group; all data in bar plots are expressed as the mean ± S.E.M. $^{*}P < 0.05$, $^{**}P < 0.01$ and $^{***}P < 0.001$ versus the HFD_C group; ns, no significance.

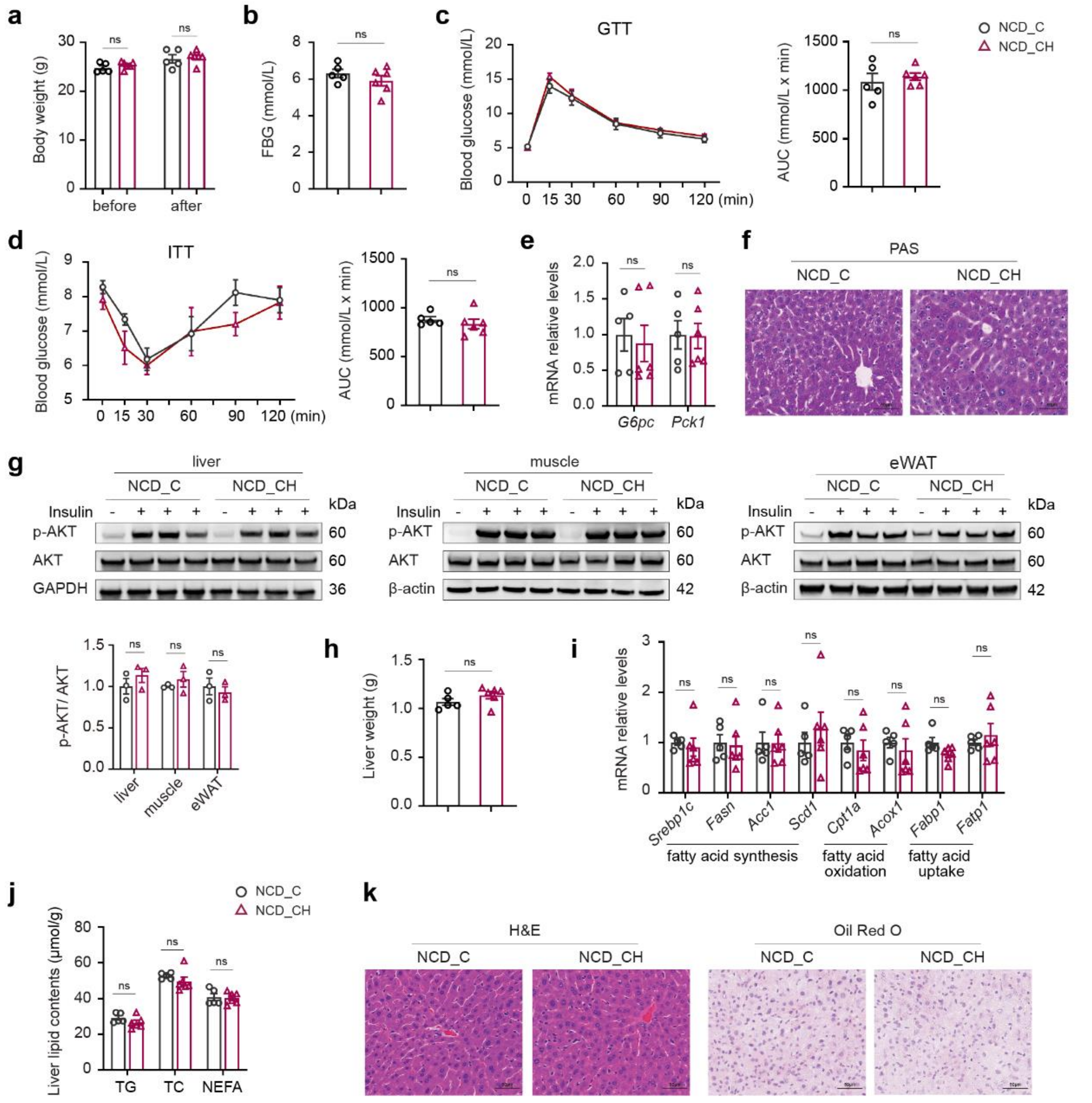

Fig. S5. CH223191 has no significant effect on hepatic insulin resistance and lipid metabolism in NCD mice.

NCD mice were treated with CH223191 at a dosage of 10 mg/kg for 10 days. (**a**) Body weight before and after CH223191 or vehicle treatment. (**b**) FBG levels of the two groups. (**c**, **d**) Blood glucose levels during the GTT and ITT, and the corresponding areas under the curves. (**e**) mRNA expression levels of G6pc and Pck1 in liver tissues. (**f**) Representative images of PAS-stained liver sections. (**g**) Expression levels of phosphorylated and total AKT in liver, muscle and iWAT. GAPDH and β-actin were used as loading controls. (**h**) Liver weight of the NCD mice treated with or without CH223191. (**i**) mRNA levels of genes regulating fatty acid metabolism in liver tissues. (**j**) TG, TC and NEFA contents in livers. (**k**) Representative images of H&E- and oil red O-stained liver sections. Scale bar, 50 μm. n=5-6 mice per group; all data in bar plots are expressed as the mean ± S.E.M. ns, no significance.

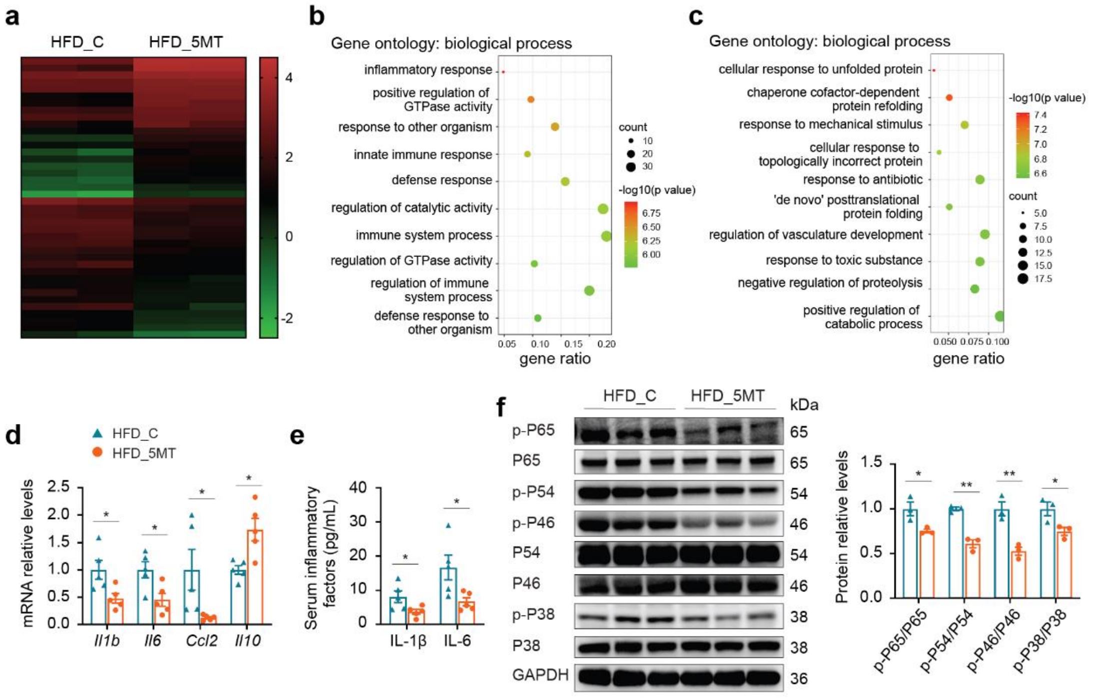

Fig. S6. 5MT mainly affects the hepatic inflammatory response in HFD mice.

(**a-c**) Total RNA was extracted from the liver tissues of HFD_C and HFD_5MT mice, and RNA sequencing and data analysis were performed. n=2 mice per group. (**a**) Heatmap showing the top 20 upregulated and top 20 downregulated genes in 5MT-treated HFD mice. (**b**, **c**) GO analysis of significantly upregulated (**b**) and downregulated (**c**) genes and the 10 highest-ranking biological process terms. (**d**) mRNA expression levels of proinflammatory factors (*Il1b*, *Il6* and *Ccl2*) and anti-inflammatory factor *Il10* in the livers of HFD mice treated with or without 5MT. (**e**) Serum levels of IL-1β and IL-6 in the indicated mice. (**f**) Protein expression levels of phosphorylated and total P65, P54/46 and P38 in the liver. GAPDH was used as the loading control. D, E, n=5 mice per group. The data in bar plots are expressed as the mean ± S.E.M. $^{*}P < 0.05$ and $^{**}P < 0.01$ versus the HFD_C group.

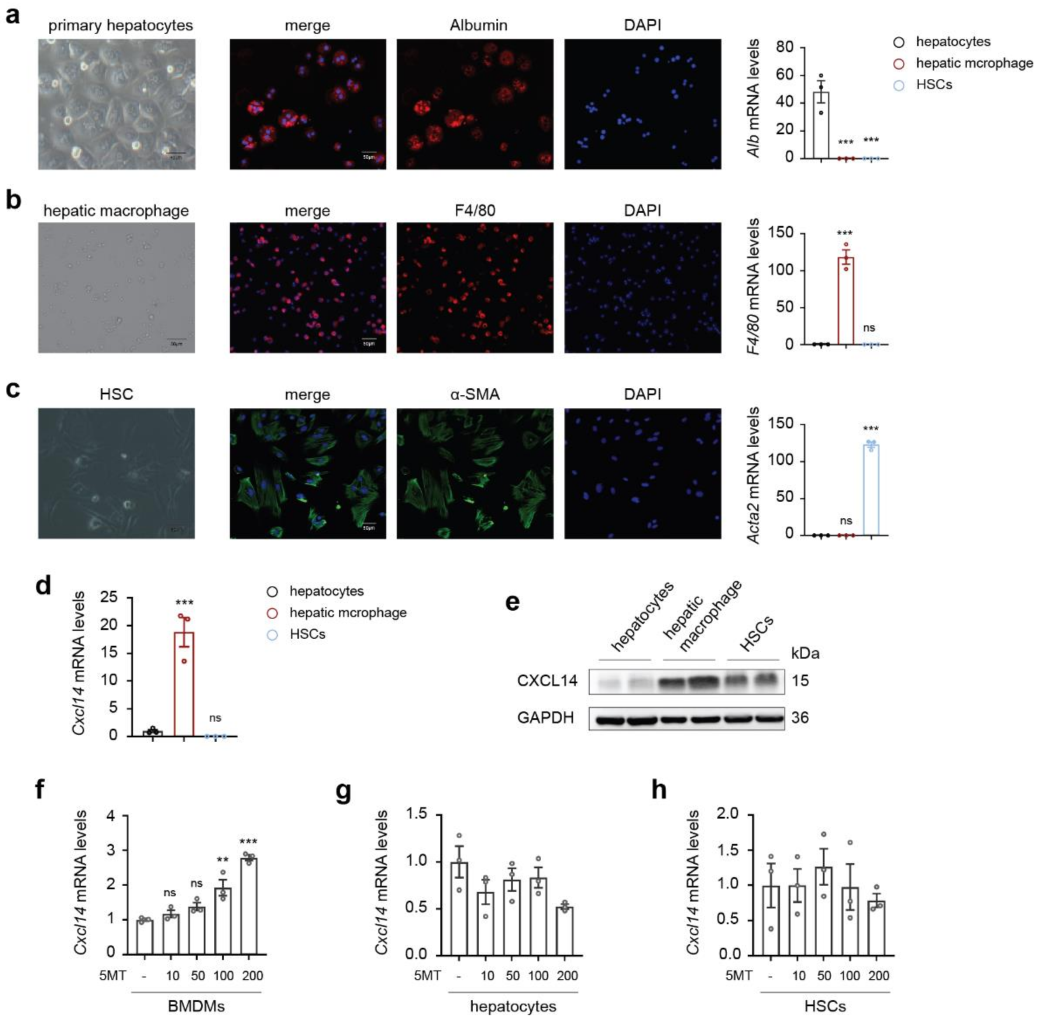

Fig. S7. The expression levels of CXCL14 in different primary cells.

Different primary cells were isolated from the livers of C57BL/6J mice (7-8 weeks). (**a-c**) IF staining and qPCR analysis of the expression levels of specific cell markers (Alb, F4/80 and α-SMA) in primary hepatocytes, hepatic macrophages and hepatic stellate cells (HSCs). Scale bar, 50 µm. (**d**, **e**) mRNA and protein expression levels of CXCL14 in different primary cells isolated from mouse liver. GAPDH was used as the loading control. (**f-h**) BMDMs, hepatocytes and HSCs were treated with 5MT (10~200 µM) for 24 hours, in the presence of 20 ng/mL LPS for 6 hours. The mRNA expression level of Cxcl14 was analyzed by qPCR. n=3, all data in bar plots are expressed as the mean ± S.E.M. $^{**}P < 0.01$ and $^{***}P < 0.001$ versus primary hepatocytes or the vehicle group; ns, no significance.

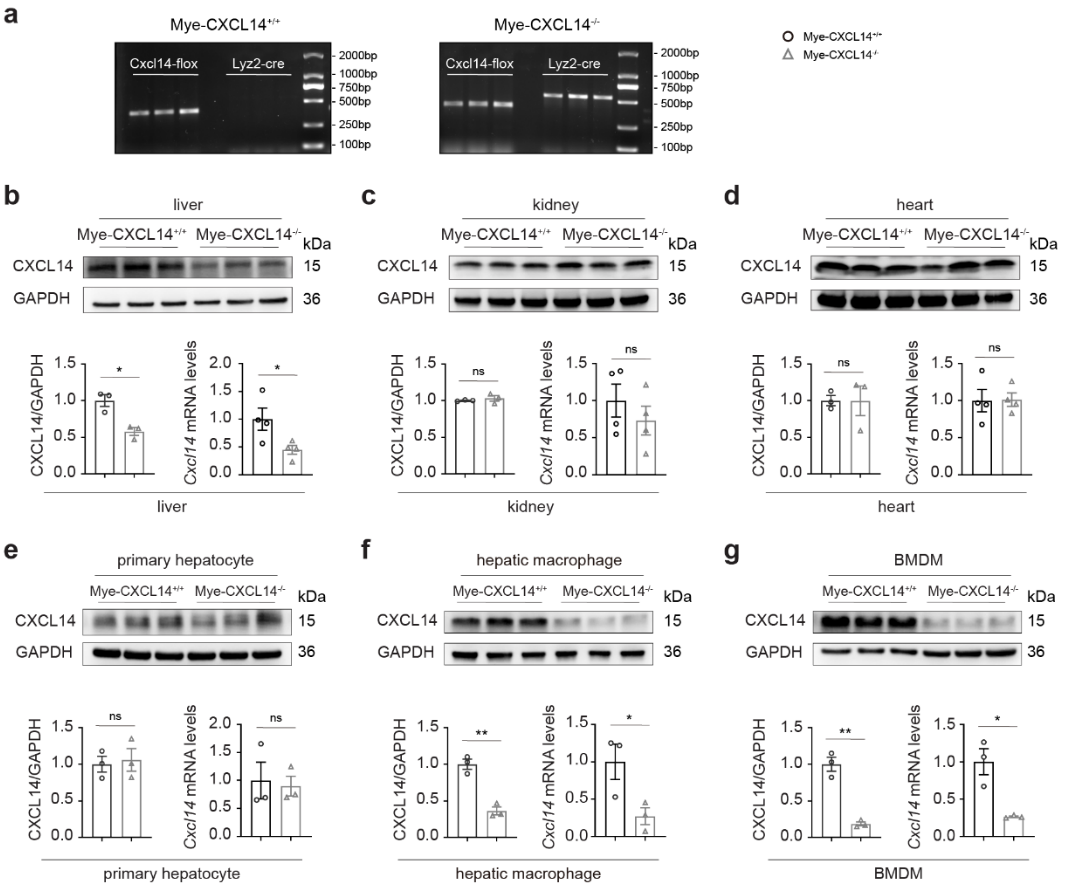

Fig. S8. Identification of myeloid-specific CXCL14 knockout mice.

(**a**) Genotyping of myeloid cell-specific CXCL14 knockout mice. (**b**-d) Protein and mRNA expression levels of CXCL14 in the liver (**b**), kidney (**c**) and heart (d) of Mye-CXCL14$^{+/+}$ and Mye-CXCL14$^{-/-}$ mice. (**e-g**) Protein and mRNA expression levels of CXCL14 in primary hepatocytes (**e**), hepatic macrophages (**f**) and BMDMs (**g**) isolated from Mye-CXCL14$^{+/+}$ and Mye-CXCL14$^{-/-}$ mice. GAPDH was used as the loading control. n=3-4, all data in bar plots are expressed as the mean ± S.E.M. $^{*}P < 0.05$ and $^{**}P < 0.01$ versus the Mye-CXCL14$^{+/+}$ group; ns, no significance.

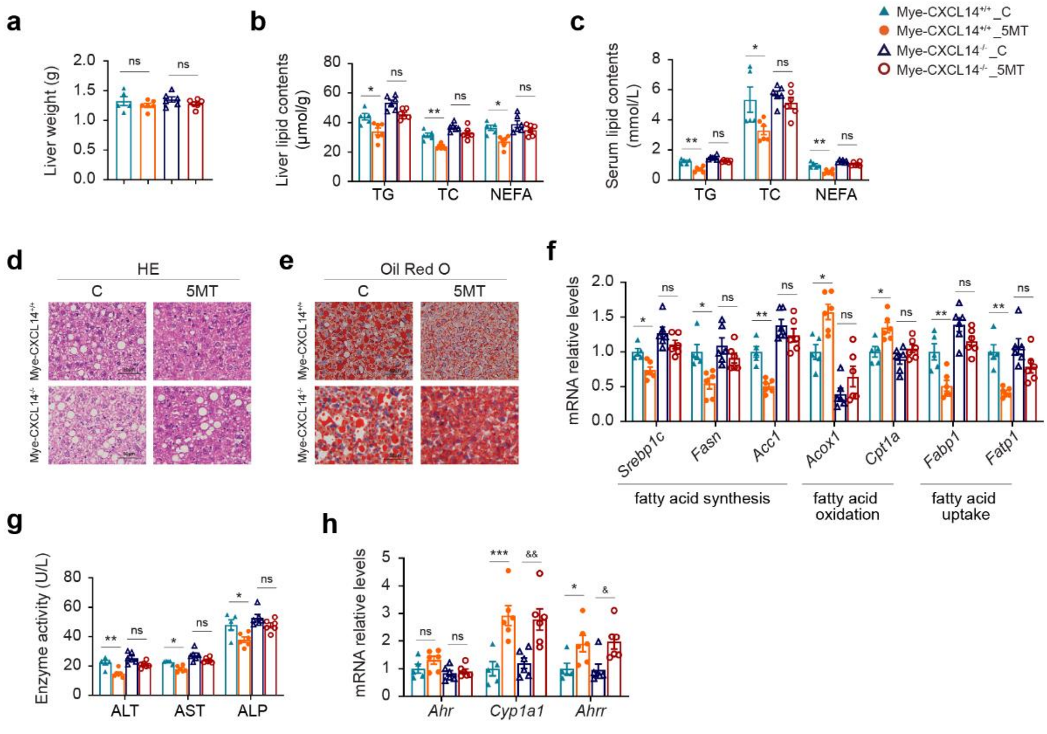

Fig. S9. 5MT improves hepatic steatosis in a macrophage CXCL14-dependent manner.

HFD-fed Mye-CXCL14$^{+/+}$ and Mye-CXCL14$^{-/-}$ mice were treated with 5MT (30 mg/kg) or vehicle for 6 weeks. (**a**) Liver weights of Mye-CXCL14$^{+/+}$ and Mye-CXCL14$^{-/-}$ mice treated with or without 5MT. (**b**, **c**) TG, TC and NEFA contents in the liver and serum. (**d**, **e**) Histological images of H&E- and oil red O-stained liver sections from the indicated mice. Scale bar, 50 μm. (**f**) mRNA levels of genes regulating fatty acid synthesis, oxidation and uptake in the liver. (**g**) Serum ALT, AST and ALP levels in each group. (**h**) mRNA expression levels of AHR target genes in the livers of each group. n=5-6 mice per group; all data in bar plots are expressed as the mean ± S.E.M. $^{*}P < 0.05$, $^{**}P < 0.01$ and $^{***}P < 0.001$ versus the Mye-CXCL14$^{+/+}$_C group; $^{\&}P < 0.05$ and $^{\&\&}P < 0.01$ versus the Mye-CXCL14$^{-/-}$_C group; ns, no significance.

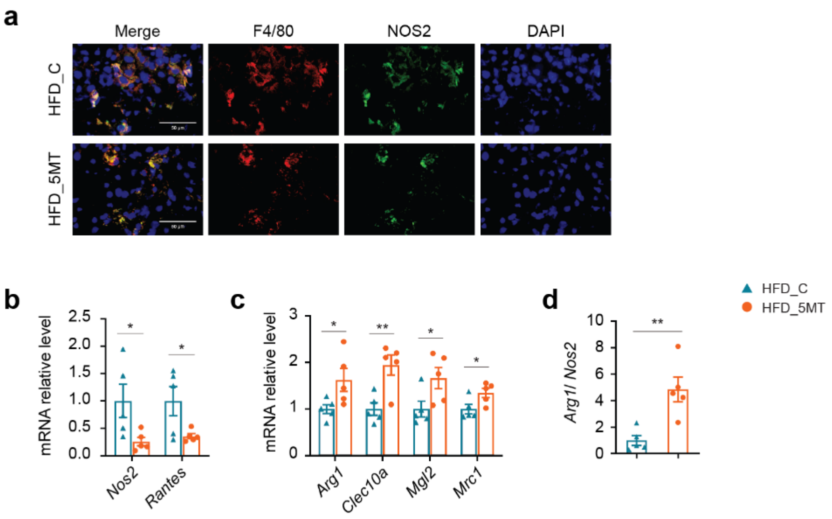

Fig. S10. 5MT reduces the number of M1 macrophages in the liver of HFD mice.

(**a**) IF staining of F4/80 and NOS2 in liver tissue sections from HFD_C and HFD_5MT mice. Scale bar, 50 μm. (**b**, **c**) mRNA expression levels of M1 (*Nos2* and *Rantes*) and M2 (Arg1, Clec10a, Mgl2 and Mrc1) related markers in the liver. (**d**) The ratio of Arg1 to Nos2 mRNA expression levels. n=5 mice per group; all data in bar plots are expressed as the mean ± S.E.M. $^{*}P < 0.05$ and $^{**}P < 0.01$ versus the HFD_C group.

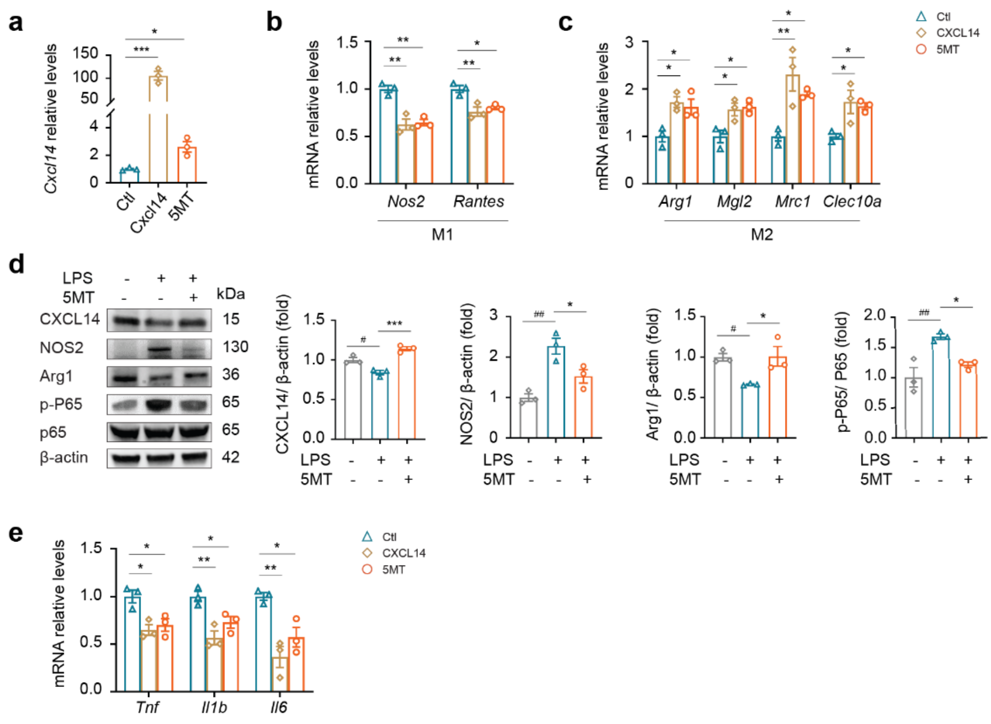

Fig. S11. 5MT regulates macrophage polarization.

(**a**) Efficiency of Cxcl14 overexpression in BMDMs. (**b**, **c**) BMDMs were transfected with a Cxcl14 overexpression plasmid or treated with 200 μM 5MT for 24 hours, and then polarized to M1 or M2 with LPS (60 ng/mL) or IL-4 (20 ng/mL). The mRNA expression levels of M1- and M2-related markers were detected by qPCR. (**d**) Protein expression levels of CXCL14, NOS2, Arg1, and phosphorylated and total P65 in treated BMDMs. β-actin was used as the loading control. (**e**) BMDMs were transfected with a Cxcl14 overexpression plasmid or treated with 5MT in the presence of LPS. mRNA expression levels of inflammatory factors (*Tnf*, *Il1b* and *Il6*) were analyzed by qPCR. n=3, all data in bar plots are expressed as the mean ± S.E.M. $^{\#}P < 0.05$ and $^{\#\#}P < 0.001$ versus the no-LPS group; $^{*}P < 0.05$, $^{**}P < 0.01$ and $^{***}P < 0.001$ versus the Ctl or LPS group.

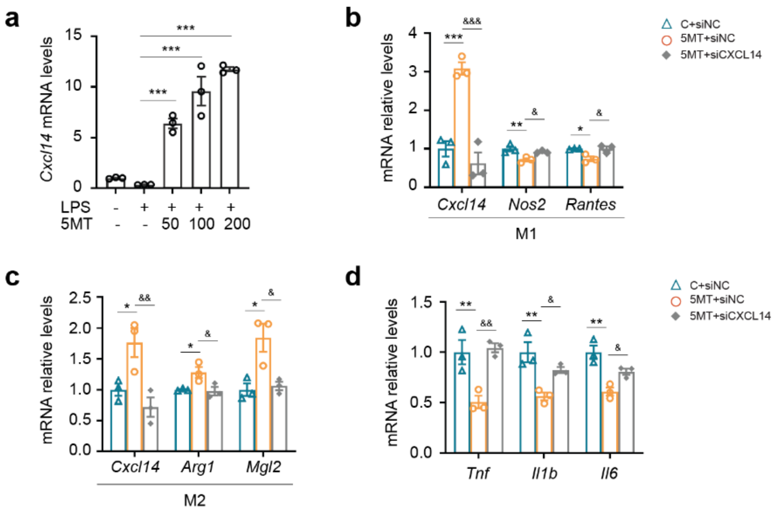

Fig. S12. 5MT regulates macrophage polarization through CXCL14 in RAW264.7 cells.

(**a**) RAW264.7 cells were treated with 5MT (50~200 μM) for 24 hours, in the presence of 20 ng/mL LPS for 6 hours. mRNA expression level of Cxcl14 was analyzed by qPCR. (**b-d**) RAW264.7 cells were transfected with siRNAs for 24 hours, and then treated with 200 μM 5MT for 24 hours. RAW264.7 cells were polarized to M1 or M2 macrophages with LPS (60 ng/mL) or IL-4 (20 ng/mL). The mRNA expression levels of Cxcl14, M1 (**b**) and M2 (**c**) related markers, and inflammatory factors (**d**) were analyzed by qPCR. n=3, all data in bar plots are expressed as the mean ± S.E.M. $^{*}P < 0.05$, $^{**}P < 0.01$ and $^{***}P < 0.001$ versus the LPS group or C+siNC group; $^{\&}P < 0.05$, $^{\&\&}P < 0.01$ and $^{\&\&\&}P < 0.001$ versus the 5MT+siNC group.

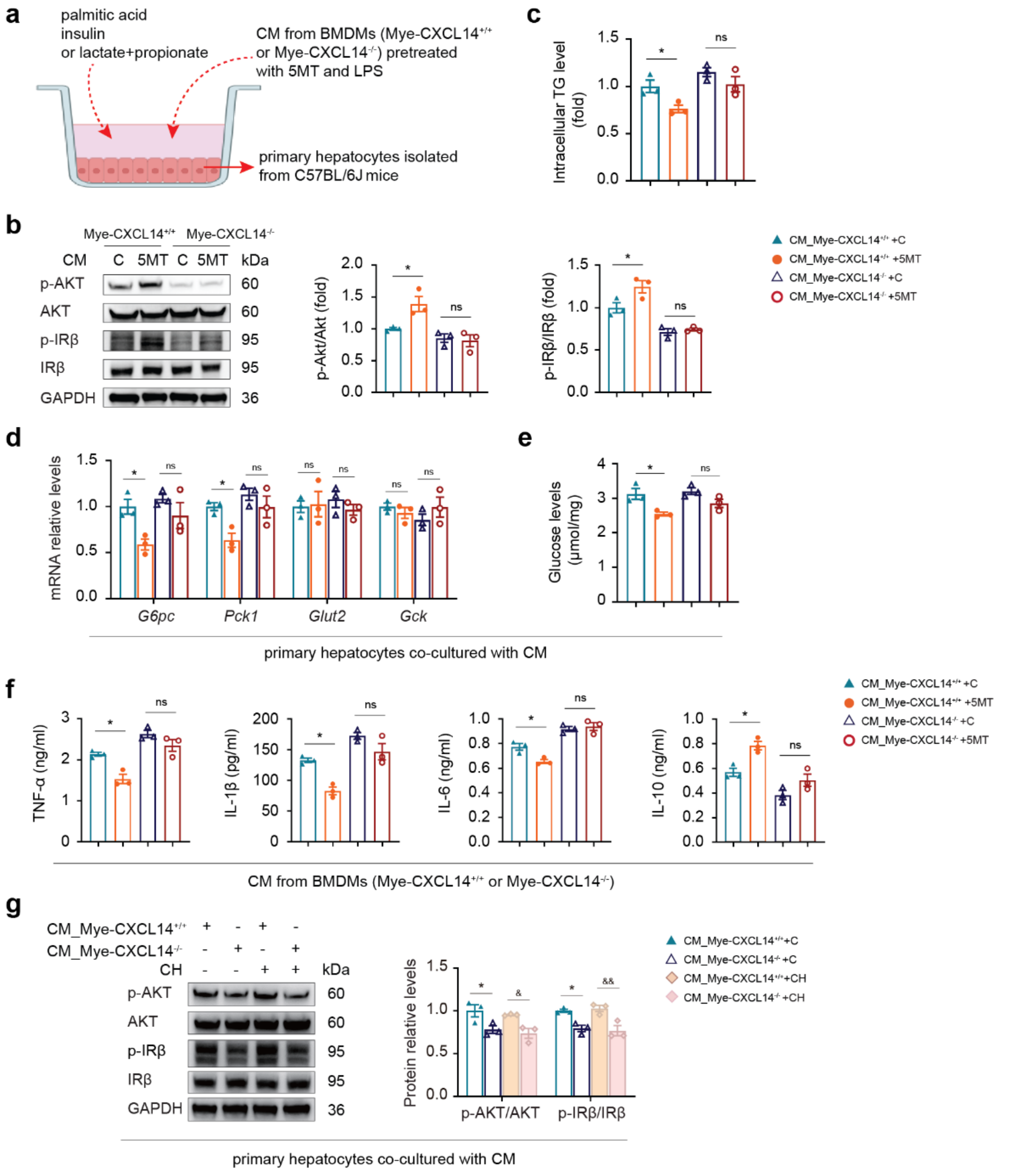

Fig. S13. 5MT activates the insulin pathway in hepatocytes in a macrophage CXCL14-dependent manner.

(**a**) Schematic showing that primary hepatocytes were cocultured with conditioned medium (CM) from Mye-CXCL14$^{+/+}$ -BMDMs or Mye-CXCL14$^{-/-}$ -BMDMs pretreated with 5MT (200 μM) and LPS (20 ng/mL). (**b**) Primary hepatocytes were incubated with CM for 24 hours, and 0.3 mM palmitic acid was added for 12 hours, followed by 100 nM insulin for 30 min. Protein expression levels of phosphorylated and total Akt and IRβ in cocultured hepatocytes. GAPDH was used as the loading control. (**c**) Intracellular TG content in cocultured hepatocytes. (**d, e**) After 24 hours of coculture, hepatocytes were incubated with lactate and propionate for 8 hours under glucose- and serum-free conditions. (**d**) mRNA expression levels of *G6pc*, *Pck1*, *Glut2*

and *Gck*. (**e**) Glucose concentrations in culture supernatant. (**f**) Concentrations of TNF-α, IL-1β, IL-6 and IL-10 in the CM from pretreated BMDMs. (**g**) Primary hepatocytes were pretreated with 15 μM CH223191 for 30 min, cocultured with CM from BMDMs pretreated with LPS (20 ng/mL), and then stimulated with insulin (100 nM) for 30mins. The protein expression levels of total and phosphorylated AKT and IRβ in cocultured hepatocytes. n=3, all data in bar plots are expressed as the mean ± S.E.M. $^{*}P < 0.05$ versus the CM_Mye-CXCL14$^{+/+}$ +C group; $^{\&}P < 0.05$ and $^{\&\&}P < 0.01$ versus the CM_Mye-CXCL14$^{+/+}$ +CH group; ns, no significance.

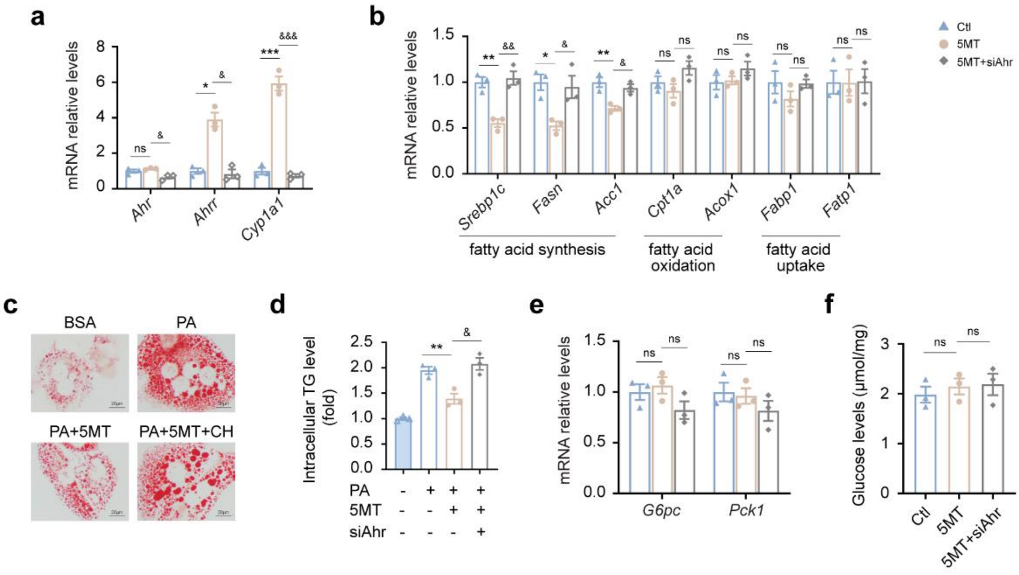

Fig. S14. 5MT inhibits fatty acid synthesis in hepatocytes via AHR.

(**a-d**) Primary hepatocytes were transfected with siAhr for 8 hours, treated with 200 μM 5MT for 24 hours, and then stimulated with 0.3mM palmitate (PA) for 12 hours. (**a**) mRNA expression levels of *Ahr*, *Ahrr* and *Cyp1a1*. (**b**) mRNA expression levels of genes regulating fatty acid synthesis, oxidation and uptake. (**c**) Intracellular lipid droplets were stained with oil red O. Scale bar, 20 μm. (**d**) TG content in treated hepatocytes. (**e-f**) 5MT-treated hepatocytes were incubated with lactate and propionate under fasting conditions 8 hours prior to harvesting. (**e**) mRNA expression levels of *G6pc* and *Pck1*. (**f**) Glucose content in the culture supernatant of hepatocytes. n=3, all data in bar plots are expressed as the mean ± S.E.M. *$P < 0.05$, **$P < 0.01$ and ***$P < 0.001$ versus the Ctl group; $^{\&}P < 0.05$, $^{\&\&}P < 0.01$ and $^{\&\&\&}P < 0.001$ versus the 5MT group; ns, no significance.

Table S1. The significantly changed genes after 5MT treatment

Gene name	FC(HFD_5MT/HFD_C)	Log2FC(HFD_5MT/HFD_C)	Pvalue	Padjust
Orm2	71.081	6.151	2.885E-70	3.522E-66
Lcn2	41.502	5.375	8.864E-65	5.410E-61
Saa1	16.228	4.020	1.053E-56	4.284E-53
Saa2	65.772	6.039	3.499E-41	1.068E-37
Hspa1b	0.147	-2.771	8.221E-33	2.007E-29
Egr1	0.019	-5.711	6.244E-27	1.270E-23
Apcs	7.108	2.830	2.037E-24	3.552E-21
Prtn3	26.202	4.712	3.366E-23	5.137E-20
Car3	0.094	-3.415	7.213E-20	9.784E-17
Slc17a8	0.113	-3.145	1.317E-18	1.608E-15
Isyna1	8.790	3.136	1.654E-17	1.835E-14
Cxcl1	0.067	-3.902	3.091E-17	3.145E-14
Moxd1	10.857	3.441	4.462E-16	4.190E-13
Cxcl14	49.684	5.635	6.110E-16	5.328E-13
Cyp3a11	0.109	-3.199	3.639E-15	2.962E-12
Hspb1	0.122	-3.037	1.336E-13	1.020E-10
Ces2c	0.163	-2.617	3.472E-13	2.493E-10
Serpinh1	0.085	-3.551	6.749E-13	4.577E-10
Ces1d	0.196	-2.354	1.198E-12	7.696E-10
P4ha1	0.233	-2.104	3.208E-11	1.958E-08
Dcn	0.251	-1.994	9.154E-11	5.322E-08
Ly6d	29.531	4.884	2.304E-10	1.279E-07
Dnajb1	0.231	-2.114	5.831E-10	3.095E-07
Dnajb4	0.284	-1.818	1.028E-09	5.231E-07
Phlda1	0.243	-2.043	1.104E-09	5.392E-07
Orm1	3.788	1.921	1.223E-09	5.744E-07
Bcl6	0.124	-3.010	1.309E-09	5.919E-07
Thbs1	0.043	-4.550	1.454E-09	6.337E-07
Fgl1	3.551	1.828	2.170E-09	9.137E-07
Ly6e	2.752	1.461	3.145E-09	1.280E-06
Rcan1	0.098	-3.345	4.255E-09	1.676E-06
Gadd45g	0.207	-2.272	7.804E-09	2.977E-06
Slc22a28	0.191	-2.391	8.896E-09	3.291E-06
Asns	11.546	3.529	1.107E-08	3.973E-06
Me1	0.248	-2.013	1.324E-08	4.617E-06
Lrg1	2.855	1.514	2.405E-08	8.156E-06
Saa3	11.997	3.585	2.876E-08	9.490E-06
Cyp2c68	0.391	-1.354	3.178E-08	1.021E-05
Ighg1	22.237	4.475	4.148E-08	1.298E-05
Hectd2os	0.264	-1.924	5.466E-08	1.668E-05

Gene name	FC(HFD_5MT/HFD_C)	Log2FC(HFD_5MT/HFD_C)	Pvalue	Padjust
Igfbp7	0.239	-2.068	7.214E-08	2.148E-05
Ecm1	0.298	-1.749	7.702E-08	2.239E-05
Gck	0.285	-1.813	8.011E-08	2.258E-05
Slc13a5	11.939	3.578	8.137E-08	2.258E-05
Rnd3	0.227	-2.136	1.054E-07	2.858E-05
Ifi27l2b	4.255	2.089	1.620E-07	4.298E-05
Cxcl10	0.084	-3.568	1.688E-07	4.385E-05
Ces1f	0.367	-1.446	1.770E-07	4.503E-05
Fzd7	0.324	-1.625	1.989E-07	4.956E-05
Igfbp1	0.146	-2.776	2.443E-07	5.966E-05
Acss3	0.099	-3.343	2.659E-07	6.364E-05
Ddit3	0.270	-1.887	3.113E-07	7.308E-05
Bag3	0.297	-1.754	3.442E-07	7.821E-05
Hsph1	0.363	-1.461	3.460E-07	7.821E-05
Igdcc4	248.330	7.956	3.660E-07	8.124E-05
Tchh	0.031	-5.020	4.053E-07	8.835E-05
Dusp6	0.281	-1.829	4.217E-07	9.031E-05
Serpina3n	2.736	1.452	5.152E-07	1.084E-04
Dnajc12	4.500	2.170	6.132E-07	1.269E-04
Acot1	0.248	-2.014	7.575E-07	1.541E-04
A630023P12Rik	12783058.188	23.608	8.073E-07	1.616E-04
Ptp4a1	0.286	-1.804	8.421E-07	1.658E-04
Izumo1r	11420163.280	23.445	9.612E-07	1.863E-04
Gpr174	11041610.652	23.396	1.012E-06	1.914E-04
Hpx	2.781	1.476	1.019E-06	1.914E-04
St8sia1	9610211.691	23.196	1.252E-06	2.271E-04
Hsp90aa1	0.437	-1.195	1.260E-06	2.271E-04
Olig1	0.120	-3.057	1.265E-06	2.271E-04
Tpm2	0.199	-2.330	1.321E-06	2.337E-04
Atf3	0.063	-3.985	1.379E-06	2.405E-04
Crybb3	15.733	3.976	1.543E-06	2.653E-04
Col3a1	0.196	-2.348	1.745E-06	2.959E-04
Sparc	0.253	-1.981	1.845E-06	3.085E-04
Tmem204	0.128	-2.963	2.089E-06	3.446E-04
Cyp4a12a	0.341	-1.551	2.234E-06	3.636E-04
Ltbp4	0.169	-2.561	2.463E-06	3.956E-04
Gnat1	3.478	1.798	2.674E-06	4.239E-04
Slc37a1	5.261	2.395	2.798E-06	4.349E-04
Akr1c20	0.381	-1.392	2.814E-06	4.349E-04
Vav1	34.723	5.118	3.102E-06	4.733E-04
Creld2	0.399	-1.327	4.498E-06	6.723E-04
Dnaja1	0.451	-1.150	4.516E-06	6.723E-04

Gene name	FC(HFD_5MT/HFD_C)	Log2FC(HFD_5MT/HFD_C)	Pvalue	Padjust
Rgs5	0.195	-2.357	4.681E-06	6.884E-04
Cpne8	5.442	2.444	5.046E-06	7.333E-04
Ces1g	0.346	-1.530	5.459E-06	7.841E-04
Orm3	6.786	2.763	6.096E-06	8.653E-04
Aldh3a2	0.432	-1.210	7.278E-06	1.021E-03
Bgn	0.330	-1.598	7.464E-06	1.035E-03
Aqp1	0.261	-1.936	8.276E-06	1.135E-03
Coq10b	0.337	-1.567	8.721E-06	1.183E-03
Abcc9	0.216	-2.212	9.741E-06	1.307E-03
Tob1	0.366	-1.449	1.044E-05	1.385E-03
Colec11	0.285	-1.809	1.089E-05	1.424E-03
Tmem71	34.457	5.107	1.096E-05	1.424E-03
Foxq1	0.257	-1.962	1.280E-05	1.645E-03
Col14a1	0.255	-1.970	1.400E-05	1.780E-03
Prelp	0.309	-1.696	1.574E-05	1.980E-03
Pnrc1	0.412	-1.280	1.960E-05	2.442E-03
Prg4	3.141	1.651	2.044E-05	2.514E-03
Dnaic1	7.071	2.822	2.059E-05	2.514E-03
Serpina12	0.400	-1.321	2.137E-05	2.583E-03
Cyp17a1	2.902	1.537	2.214E-05	2.650E-03
Dnase1l3	0.256	-1.965	2.299E-05	2.699E-03
Itih3	2.894	1.533	2.299E-05	2.699E-03
Hp	2.117	1.082	2.410E-05	2.801E-03
Manf	0.474	-1.077	2.666E-05	3.070E-03
Trp53inp1	0.314	-1.670	2.693E-05	3.072E-03
BE692007	66.506	6.055	3.191E-05	3.607E-03
Ces1e	0.337	-1.567	3.524E-05	3.920E-03
Rab11fip4	3.945	1.980	3.538E-05	3.920E-03
Rgs14	96.258	6.589	3.564E-05	3.920E-03
Zfp36l1	0.435	-1.199	3.695E-05	4.027E-03
Dusp1	0.302	-1.730	3.740E-05	4.040E-03
Osgin1	2.659	1.411	3.776E-05	4.043E-03
Reln	0.236	-2.080	3.890E-05	4.129E-03
Ptpn18	29.609	4.888	4.006E-05	4.216E-03
Sdf2l1	0.468	-1.095	4.042E-05	4.218E-03
Fam13a	0.340	-1.555	4.145E-05	4.288E-03
Hspa5	0.438	-1.190	4.206E-05	4.315E-03
Sh3bp2	3.277	1.713	4.269E-05	4.319E-03
Rgs3	2.733	1.451	4.281E-05	4.319E-03
Inhbb	0.019	-5.700	4.844E-05	4.847E-03
Thrsp	0.280	-1.838	5.158E-05	5.119E-03
Rad51b	5.556	2.474	5.824E-05	5.734E-03

Gene name	FC(HFD_5MT/HFD_C)	Log2FC(HFD_5MT/HFD_C)	Pvalue	Padjust
Trib1	0.263	-1.928	5.996E-05	5.856E-03
Arhgap27	29.732	4.894	6.080E-05	5.891E-03
Serpine1	0.119	-3.075	6.298E-05	6.054E-03
Mfng	36.359	5.184	6.367E-05	6.064E-03
P2rx7	9.021	3.173	6.408E-05	6.064E-03
Cyp2c67	0.454	-1.139	6.529E-05	6.131E-03
Cntnap1	5.444	2.445	6.691E-05	6.235E-03
Zc3h12d	4.645	2.216	6.797E-05	6.286E-03
Herpud1	0.323	-1.632	6.916E-05	6.348E-03
Gm20605	13.577	3.763	7.195E-05	6.555E-03
Hsp90ab1	0.527	-0.924	7.357E-05	6.653E-03
Pfkfb3	0.235	-2.088	7.651E-05	6.868E-03
Mpeg1	2.373	1.247	7.832E-05	6.979E-03
Ighg2b	5.872	2.554	8.145E-05	7.206E-03
Gvin1	4.590	2.198	9.044E-05	7.943E-03
Mme	0.243	-2.041	9.695E-05	8.399E-03
Arhgap15	17.505	4.130	9.700E-05	8.399E-03
Cyp2c69	0.004	-7.848	9.782E-05	8.410E-03
Evi2	39.763	5.313	9.928E-05	8.475E-03
Smpd3	5.613	2.489	1.023E-04	8.675E-03
Scn5a	119.778	6.904	1.058E-04	8.904E-03
Mpzl2	0.450	-1.151	1.079E-04	8.999E-03
Was	37.649	5.235	1.084E-04	8.999E-03
Fabp1	0.532	-0.911	1.154E-04	9.523E-03
Vamp1	16.801	4.070	1.167E-04	9.563E-03
Rab37	251.002	7.972	1.226E-04	9.949E-03
Rhob	0.335	-1.576	1.231E-04	9.949E-03
Patz1	11.892	3.572	1.239E-04	9.949E-03
Vmn2r97	23.638	4.563	1.260E-04	1.006E-02
Stip1	0.526	-0.928	1.391E-04	1.102E-02
Ccl2	0.024	-5.370	1.436E-04	1.127E-02
Timp3	0.087	-3.522	1.440E-04	1.127E-02
Plk3	0.215	-2.215	1.477E-04	1.149E-02
Inmt	0.359	-1.478	1.508E-04	1.161E-02
Ccl7	0.005	-7.748	1.512E-04	1.161E-02
Ces2d-ps	0.210	-2.254	1.610E-04	1.229E-02
Hspe1	0.525	-0.929	1.630E-04	1.230E-02
Maff	0.158	-2.658	1.632E-04	1.230E-02
Golm1	6.320	2.660	1.686E-04	1.262E-02
Fgr	10.100	3.336	1.710E-04	1.273E-02
Lum	0.139	-2.848	1.794E-04	1.328E-02
Icos	218.765	7.773	1.820E-04	1.338E-02

Gene name	FC(HFD_5MT/HFD_C)	Log2FC(HFD_5MT/HFD_C)	Pvalue	Padjust
Adgre5	10.014	3.324	1.869E-04	1.366E-02
Pld4	4.383	2.132	1.895E-04	1.377E-02
Helz2	2.185	1.127	1.929E-04	1.393E-02
Mup21	0.460	-1.119	2.030E-04	1.458E-02
Ppm1h	13.540	3.759	2.094E-04	1.489E-02
Ms4a4c	66.640	6.058	2.101E-04	1.489E-02
Slc43a3	0.311	-1.684	2.125E-04	1.489E-02
Apobec3	20.881	4.384	2.130E-04	1.489E-02
Cyp2c40	0.263	-1.926	2.135E-04	1.489E-02
Sacs	30.078	4.911	2.153E-04	1.494E-02
Slc38a2	0.442	-1.178	2.245E-04	1.549E-02
Jak3	4.913	2.297	2.279E-04	1.557E-02
Elmo1	14.912	3.898	2.284E-04	1.557E-02
Rpap3	3.036	1.602	2.295E-04	1.557E-02
Tm4sf20	88.595	6.469	2.396E-04	1.616E-02
Iqgap1	10.607	3.407	2.412E-04	1.618E-02
Btg3	4.807	2.265	2.440E-04	1.628E-02
Csf1r	3.229	1.691	2.502E-04	1.660E-02
Clcf1	70.880	6.147	2.524E-04	1.665E-02
Rapgef5	0.124	-3.013	2.596E-04	1.704E-02
Aldh1a1	0.477	-1.069	2.620E-04	1.710E-02
Itga6	25.031	4.646	2.646E-04	1.718E-02
Tff3	2.609	1.384	2.698E-04	1.743E-02
Hspa4l	0.417	-1.264	2.751E-04	1.767E-02
Gm26740	58.752	5.877	2.894E-04	1.844E-02
Atp10a	18.414	4.203	2.901E-04	1.844E-02
Epsti1	17.504	4.130	3.004E-04	1.892E-02
Kcnh6	8.915	3.156	3.006E-04	1.892E-02
Pam	0.283	-1.819	3.074E-04	1.924E-02
Tagap	31.219	4.964	3.099E-04	1.931E-02
Adamts1	0.235	-2.091	3.119E-04	1.933E-02
Ttc39a	11.496	3.523	3.210E-04	1.979E-02
Col15a1	4.809	2.266	3.256E-04	1.997E-02
Cd36	0.223	-2.166	3.296E-04	2.012E-02
Pdia4	0.503	-0.991	3.358E-04	2.031E-02
Me2	16.764	4.067	3.360E-04	2.031E-02
Fitm1	0.306	-1.709	3.415E-04	2.053E-02
Hpgd	0.456	-1.131	3.485E-04	2.085E-02
Krt23	0.020	-5.651	3.501E-04	2.085E-02
Nemp2	68.321	6.094	3.524E-04	2.088E-02
Klhl6	24.016	4.586	3.579E-04	2.103E-02
Hspa1a	0.128	-2.962	3.583E-04	2.103E-02

Gene name	FC(HFD_5MT/HFD_C)	Log2FC(HFD_5MT/HFD_C)	Pvalue	Padjust
Selenbp2	0.356	-1.489	3.657E-04	2.136E-02
Ighg2c	6.210	2.635	3.702E-04	2.142E-02
Cacybp	0.505	-0.985	3.703E-04	2.142E-02
Oas3	33.918	5.084	3.731E-04	2.148E-02
Scara5	5.380	2.427	3.768E-04	2.160E-02
Itih4	2.519	1.333	3.805E-04	2.162E-02
Klf10	0.320	-1.646	3.819E-04	2.162E-02
Tmc8	30.848	4.947	3.825E-04	2.162E-02
Arrdc1	21.983	4.458	3.886E-04	2.186E-02
Nin	19.250	4.267	3.964E-04	2.212E-02
Akr1c14	0.371	-1.430	3.969E-04	2.212E-02
Cd5l	2.609	1.383	3.987E-04	2.212E-02
Kcnt2	6.714	2.747	4.025E-04	2.224E-02
Plvap	0.330	-1.599	4.080E-04	2.229E-02
Syt12	10.889	3.445	4.088E-04	2.229E-02
Atp10d	15.614	3.965	4.096E-04	2.229E-02
Gbp8	16.376	4.034	4.109E-04	2.229E-02
Gm10053	0.476	-1.072	4.165E-04	2.241E-02
Hspa8	0.562	-0.832	4.167E-04	2.241E-02
Rgs16	0.201	-2.315	4.229E-04	2.264E-02
Dmgdh	0.452	-1.144	4.263E-04	2.273E-02
Ntn1	0.188	-2.412	4.319E-04	2.292E-02
Gstt1	0.436	-1.196	4.359E-04	2.302E-02
Crybg1	2.190	1.131	4.375E-04	2.302E-02
Angptl3	0.430	-1.218	4.412E-04	2.312E-02
Ablim1	6.993	2.806	4.435E-04	2.314E-02
Nfkbia	0.505	-0.985	4.507E-04	2.331E-02
Gm29216	0.523	-0.936	4.507E-04	2.331E-02
mt-Nd4l	0.438	-1.191	4.646E-04	2.393E-02
Stat4	32.205	5.009	4.728E-04	2.425E-02
Ighm	10.425	3.382	4.810E-04	2.453E-02
Cd226	52.336	5.710	4.841E-04	2.453E-02
Ly6c2	24.219	4.598	4.843E-04	2.453E-02
Col27a1	0.250	-2.002	4.911E-04	2.477E-02
Gpat3	3.509	1.811	4.932E-04	2.478E-02
Tmem154	50.376	5.655	5.029E-04	2.516E-02
C1qb	2.542	1.346	5.079E-04	2.531E-02
Tnfrsf18	88.398	6.466	5.105E-04	2.534E-02
Sult1b1	0.413	-1.275	5.227E-04	2.569E-02
Igkc	4.209	2.074	5.229E-04	2.569E-02
Prex1	9.733	3.283	5.241E-04	2.569E-02
Tmem39a	0.425	-1.236	5.402E-04	2.638E-02

Gene name	FC(HFD_5MT/HFD_C)	Log2FC(HFD_5MT/HFD_C)	Pvalue	Padjust
Adam19	37.423	5.226	5.496E-04	2.669E-02
Angptl6	0.324	-1.625	5.510E-04	2.669E-02
Ly75	10.214	3.352	5.647E-04	2.723E-02
Dennd1c	13.841	3.791	5.667E-04	2.723E-02
Gm4070	5.259	2.395	5.688E-04	2.723E-02
Lipg	0.289	-1.790	5.955E-04	2.805E-02
Tmem268	3.566	1.834	5.958E-04	2.805E-02
Nnmt	2.080	1.057	5.960E-04	2.805E-02
Jade2	5.259	2.395	5.973E-04	2.805E-02
Cyb561	2.878	1.525	5.974E-04	2.805E-02
Noct	0.319	-1.647	6.001E-04	2.807E-02
Sucnr1	0.373	-1.421	6.178E-04	2.871E-02
Ucp2	4.743	2.246	6.185E-04	2.871E-02
Nlrc5	5.792	2.534	6.608E-04	3.049E-02
Rasgef1b	0.220	-2.183	6.618E-04	3.049E-02
Ccn1	0.165	-2.603	6.648E-04	3.051E-02
Zbp1	5.065	2.341	6.761E-04	3.091E-02
Rnf144a	4.866	2.283	6.799E-04	3.093E-02
Gm18853	183.382	7.519	6.836E-04	3.093E-02
Tsc22d1	0.287	-1.802	6.840E-04	3.093E-02
Runx1	33.491	5.066	6.908E-04	3.112E-02
Pygb	9.631	3.268	6.959E-04	3.123E-02
Mthfd1l	17.359	4.118	7.027E-04	3.132E-02
Amy1	0.439	-1.188	7.058E-04	3.132E-02
Acaa1b	0.499	-1.003	7.080E-04	3.132E-02
Rbm3	2.946	1.559	7.080E-04	3.132E-02
Art2b	42.718	5.417	7.134E-04	3.144E-02
Gmip	11.005	3.460	7.187E-04	3.156E-02
Sla	17.832	4.156	7.314E-04	3.200E-02
Sparcl1	0.139	-2.850	7.374E-04	3.215E-02
Cdc25b	24.275	4.601	7.439E-04	3.221E-02
Rbl1	14.047	3.812	7.439E-04	3.221E-02
Palld	0.447	-1.163	7.481E-04	3.227E-02
Nxpe3	39.436	5.301	7.587E-04	3.261E-02
Ssbp4	6.129	2.616	7.716E-04	3.305E-02
Agap2	8.254	3.045	7.778E-04	3.312E-02
Lamb1	0.187	-2.421	7.787E-04	3.312E-02
Acta2	0.131	-2.933	7.900E-04	3.349E-02
Mical1	17.471	4.127	8.005E-04	3.381E-02
Mbd1	0.329	-1.606	8.184E-04	3.445E-02
Pitpnm1	11.180	3.483	8.230E-04	3.453E-02
Oas2	14.539	3.862	8.291E-04	3.466E-02

Gene name	FC(HFD_5MT/HFD_C)	Log2FC(HFD_5MT/HFD_C)	Pvalue	Padjust
Gsta3	0.427	-1.228	8.440E-04	3.517E-02
Cyp51	0.549	-0.864	8.545E-04	3.548E-02
Emilin1	0.320	-1.646	8.775E-04	3.631E-02
Siglec1	6.888	2.784	8.848E-04	3.649E-02
Tubb2a	0.442	-1.177	8.933E-04	3.672E-02
Zfp36	0.360	-1.473	9.218E-04	3.776E-02
P2ry10	28.489	4.832	9.265E-04	3.783E-02
Arhgef2	14.749	3.883	9.405E-04	3.827E-02
Uba7	6.670	2.738	9.464E-04	3.830E-02
Hmgcr	0.483	-1.049	9.475E-04	3.830E-02
Gadd45a	0.232	-2.107	9.654E-04	3.877E-02
Acap1	81.808	6.354	9.655E-04	3.877E-02
Igfbp3	0.289	-1.791	1.000E-03	4.004E-02
Hyou1	0.498	-1.005	1.027E-03	4.098E-02
Slc22a30	0.402	-1.316	1.035E-03	4.115E-02
Nt5c3b	9.038	3.176	1.062E-03	4.208E-02
Slc2a3	40.216	5.330	1.067E-03	4.216E-02
Ephb6	15.222	3.928	1.081E-03	4.259E-02
Gimap1	16.233	4.021	1.111E-03	4.361E-02
Cd9	2.207	1.142	1.114E-03	4.361E-02
Ces2e	0.467	-1.099	1.133E-03	4.420E-02
Rasgrp2	5.056	2.338	1.143E-03	4.443E-02
Foxp3	56.330	5.816	1.173E-03	4.538E-02
Ano6	3.990	1.996	1.175E-03	4.538E-02
Tlr1	34.177	5.095	1.189E-03	4.578E-02
4931406C07Rik	0.496	-1.011	1.204E-03	4.622E-02
Slc35b3	2.879	1.526	1.220E-03	4.668E-02
Plec	3.221	1.687	1.223E-03	4.668E-02
Gpsm3	13.283	3.732	1.242E-03	4.718E-02
Dennd2d	9.348	3.225	1.250E-03	4.718E-02
Cst7	188.759	7.560	1.252E-03	4.718E-02
A430078G23Rik	55.073	5.783	1.252E-03	4.718E-02
Rhoh	55.031	5.782	1.263E-03	4.743E-02
Lhfp	0.190	-2.392	1.274E-03	4.760E-02
Slc14a1	121.441	6.924	1.275E-03	4.760E-02
Dusp2	26.425	4.724	1.284E-03	4.779E-02
Mvp	1.865	0.899	1.294E-03	4.802E-02
Ccdc117	0.516	-0.956	1.300E-03	4.810E-02
Arl5b	0.478	-1.065	1.336E-03	4.927E-02

Table S2. List of reagents and kits

Reagent/kit	Company	Cat No.
5-methoxytrptamine	Sigma-Aldrich	#286583
CH-223191	Selleck	#S7711
Recombinant M-CSF	SinoBiological	#51112-MNAH
Lipopolysaccharides	MedChemExpress	#HY-D1056
Recombinant IL-4	SinoBiological	#51084-MNAE
Mouse IL-1β ELISA Kit	Multi Sciences	#EK201B/3
Mouse IL-6 ELISA Kit	Multi Sciences	#EK206/3
Mouse IL-10 ELISA Kit	Multi Sciences	#EK210/4
Mouse TNFα ELISA Kit	Multi Sciences	#EK282
Rat/Mouse Insulin ELISA Kit	Millipore	#EZRMI-13K
EZ-Magna ChIP™ A/G ChIP Kit	Millipore	#17-10086
Dual-luciferase reporter assay system	Promega	#E1910
Periodic Acid Schiff Stain Kit	Solarbio	#G1281
Oil Red O	Sigma-Aldrich	#O0625
Cytoplasmic and Nuclear Protein Extraction Kit	Beyotime	#P0027
Lactic Acid assay kit	NanJing Jiancheng Bioengineering Institute	#A019-2-1
Triglyceride assay kit	Applygen	#E1013
Total cholesterol assay kit	NanJing Jiancheng Bioengineering Institute	#A111-1-1
Nonesterified Free fatty acids assay kit	NanJing Jiancheng Bioengineering Institute	#A042-2-1
Alanine aminotransferase Assay Kit	NanJing Jiancheng Bioengineering Institute	#C009-2-1
Aspartate aminotransferase Assay Kit	NanJing Jiancheng Bioengineering Institute	#C010-2-1
Alkaline phosphatase Assay Kit	NanJing Jiancheng Bioengineering Institute	#A059-2-2
Microalbumin Assay Kit	NanJing Jiancheng Bioengineering Institute	#H127-1-2
Creatinine Assay Kit	NanJing Jiancheng Bioengineering Institute	#C011-2-1
Oleic acid	Sigma-Aldrich	#364525
Palmitic acid	Sigma-Aldrich	#P0500
Insulin	MedChemExpress	#HY-P1156
Seahorse XFp FluxPak	Agilent Technologies	#103022-100

**Reagent/kit**	**Company**	**Cat No.**
1.0M glucose	Agilent Technologies	#103577-100
100mM pyruvate	Agilent Technologies	#103578-100
200mM glutamine	Agilent Technologies	#103579-100
Type IV collagenase	Sigma-Aldrich	#C4-22-1G
Oligomycin	MedChemExpress	#HY-N6782
FCCP	MedChemExpress	#HY-100410
Rotenone	MedChemExpress	#HY-B1756
2-Deoxy-D-glucose	MedChemExpress	#HY-13966
Hanks' Balanced Salt Solution	Beyotime	#C0218
Hanks' Balanced Salt Solution (with Ca^{2+})	Beyotime	#C0219
Collagen type I	Solarbio	#C8065
Percoll	Solarbio	#P8370
Dexamethasone	MedChemExpress	#HY-14648
Poly-L-lysine	Beyotime	#C0313
MaM macrophage medium	ScienCell Research Laboratories	#1921
Penicillin-Streptomycin Solution	HyClone	#SV30010
Fetal bovine serum	Gibco	#A3161002C
Cell Counting Kit-8	Selleck	#B34302
Pronase	Roche	#10165921001
Protease inhibitors	Roche	#11836153001
M-PER™ Mammalian Protein Extraction Reagent	Thermo Scientific	#78501
Protease Inhibitor Cocktail	MedChemExpress	#HY-K0010
Phosphatase Inhibitor Cocktail II	MedChemExpress	#HY-K0022
Zombie NIR™ Fixable Viability Kit	BioLegend	#423105
Normal chow diet	Beijing HFK Bioscience	#1015
High fat diet	Research Diets	#D12492
Lactic acid	MedChemExpress	#HY-B2227
Pyruvic acid sodium	MedChemExpress	#HY-W015913
Glucose Content Detection Kit	APPLYGEN	#E1011

Table S3. List of antibodies

Antibodies	Company	Cat No.
anti-p-Akt for WB	Cell Signaling Technology	#4060
anti-Akt for WB	Cell Signaling Technology	#4691
anti-p-IRβ for WB	Cell Signaling Technology	#3021
anti-IRβ for WB	Cell Signaling Technology	#3025
anti-p-GSK-3β for WB	Cell Signaling Technology	#9322
anti-GSK-3β for WB	Cell Signaling Technology	#9315S
anti-p-P65 for WB	Cell Signaling Technology	#3033
anti-P65 for WB	Cell Signaling Technology	#8242
anti-p-JNK for WB	Cell Signaling Technology	#4668
anti-JNK for WB	Cell Signaling Technology	#9252
anti-p-P38 for WB	Cell Signaling Technology	#4511
anti-P38 for WB	Cell Signaling Technology	#9228
anti-CXCL14 for WB	Novus Biologicals	#NBP1-31398
anti-AhR for WB/IF	Proteintech	#17840-1-AP
anti-LaminB1 for WB	Proteintech	#66095-1-Ig
anti-NOS2 for WB	Cell Signaling Technology	#13120
anti-Arg1 for WB	Cell Signaling Technology	#93668
anti-NOS2 for IF	Proteintech	#18985-1-AP
anti-F4/80 for IF	Cell Signaling Technology	#71299
anti-Albumin for IF	Proteintech	#66051-1-Ig
anti-α-SMA for IF	Cell Signaling Technology	#19245
anti-CD31 for IF	Proteintech	#28083-1-AP
anti-Cytokeratin 19 for IF	Proteintech	#10712-1-AP
anti-β-Actin for WB	Santa Cruz Biotechnology	#sc-47778
anti-GAPDH for WB	Santa Cruz Biotechnology	#sc-20357
anti-mouse CD16/32	BioLegend	#101319
AF488 anti-mouse F4/80	BioLegend	#123120
Pacific Blue anti-mouse CD11c	BioLegend	#117322
AF647 anti-mouse CD206	BD Biosciences	#565250
PPARα	Proteintech	#66826-1-Ig
SREBP1	Proteintech	#14088-1-AP

Table S4. Sequences of siRNA and primers (for mouse)

Gene	siRNA/primer	Sequence (5' - 3')
Cxcl14 siRNA	sense	CUGCGAGGAGAAGAUGGUUAUTT
	antisense	AUAACCAUCUUCUCCUCGCAGTT
Ahr siRNA	sense	GCUGGAUAAUUCAUCUGGUUUTT
	antisense	AAACCAGAUGAAUUAUCCAGCTT
Cxcl14_promoter-1	forward	GCCTGACCTTCTTCCAAGG
	reverse	CACACACGGACACACTCACA
Cxcl14_promoter-2	forward	TGTGAGTGTGTCCGTGTGTG
	reverse	TATGCAGAACCACTCGGTGA
Cxcl14-Flox-1	forward	GCCTTTGGGCTGTGTTACTATG
	reverse	AGGCAGAAGGTTGGATGTCATG
Cxcl14-Flox-2	forward	GCATCGCATTGTCTGAGTAGGTG
	reverse	ACTGTGCCCTGTACCTTCATAGC
Lyz2-iCre-1	forward	AGTGCTGAAGTCCATAGATCGG
	reverse	CTGATTCTCCTCATCACCAGG
Lyz2-iCre-1	forward	AGTGCTGAAGTCCATAGATCGG
	reverse	GTCACTCACTGCTCCCCTGT
DRE-1	forward	GCCTGACCTTCTTCCAAGG
	reverse	CACACACGGACACACTCACA
DRE-2	forward	TGTGAGTGTGTCCGTGTGTG
	reverse	TATGCAGAACCACTCGGTGA
Cxcl14	forward	GAAGATGGTTATCGTCACCACC
	reverse	CGTTCCAGGCATTGTACCACT
Srebp1c	forward	CACTTCTGGAGACATCGCAAAC
	reverse	ATGGTAGACAACAGCCGCATC
Fasn	forward	AAGTTGCCCGAGTCAGAGAA
	reverse	GACCGCTTGGGTAATCCATA
Acc1	forward	CGAAGGGCTTACATTGCCTA
	reverse	GGATGTTCCCTCTGTTTGGA
Scd1	forward	CATCATTCTCATGGTCCTGCT
	reverse	CCCAGTCGTACACGTCATTTT
Cpt1a	forward	TCAAGCCAGACGAAGAACATC
	reverse	TGGTAGGAGAGCAGCACCTT
Acox1	forward	TTCTCAACAGCCCAACTGTG
	reverse	GGCATGTAACCCGTAGCACT
Fabp1	forward	GGAAGGACATCAAGGGGGTG
	reverse	TCACCTTCCAGCTTGACGAC

Gene	siRNA/primer	Sequence (5' - 3')
Fatp1	forward	ACTCTGCAAAGGGCTCATCC
	reverse	GCACGCATGCTGTAGGAATG
G6pc	forward	GCGCTTGGATTCTACCTGCT
	reverse	GAGGCTGGCAAAGGGTGTAG
Pck1	forward	GGGTGGAAGGTCGAATGTGT
	reverse	TCTTCACTGAGGTGCCAGGA
Tnf-a	forward	ACTGGCAGAAGAGGCACTCC
	reverse	GCCACAAGCAGGAATGAGAA
Il-1b	forward	CCAGGATGAGGACATGAGCA
	reverse	CGGAGCCTGTAGTGCAGTTG
Il-6	forward	TCCATCCAGTTGCCTTCTTG
	reverse	AAGCCTCCGACTTGTGAAGTG
Ccl2	forward	ACAAGAGGATCACCAGCAGC
	reverse	GGACCCATTCCTTCTTGGGG
Il-10	forward	AGGCGCTGTCATCGATTTCT
	reverse	AGGAAGAACCCCTCCCATCA
Ahr	forward	CGCGGGCACCATGAGCAG
	reverse	GAGACTCAGCTCCTGGATGG
Cyp1a1	forward	GTCAGGACAGGAAGCTGGAC
	reverse	GATAGGGCAGCTGAGGTCTG
Ahrr	forward	AGCCCTGTCACCTGAAGAAC
	reverse	ATGGCCCAGTGTAGCTGTCT
Nos2	forward	GCCCAGGAGGAGAGAGAT
	reverse	GCAAAGAGGACTGTGGCT
Rantes	forward	TGCTTTGCCTACCTCTCC
	reverse	CACACACTTGGCGGTTC
Arg1	forward	ACGGTCTGTGGGGAAAG
	reverse	TCAGGGGAGTGTTGATGTC
Clec10a	forward	GGATGGGACCGACTTTGAGA
	reverse	CTTGGCCAGCTTCATCTCAC
Mgl2	forward	CCTCTCTTGGACCCACCT
	reverse	ATGACCACCAGTAGCAGGA
Mrc1	forward	TTGTGGAGCAGATGGAAGGT
	reverse	TTCCACACCAGAGCCATCC
Hk2	forward	GGGCTAGGAGCTACCACACA
	reverse	GGTCCAGAGCCAGGAACTC
Pkm	forward	GGTGGCTCTGGATACAAAGGG
	reverse	ACTTCTCCATGTAAGCGTTGTC

Gene	siRNA/primer	Sequence (5' - 3')
Ldha	forward	GAGTGGTGTGAATGTTGCCG
	reverse	CACCTCGTAGGCACTGTCCA
Atp5a1	forward	CATTGGTGATGGTATTGCGC
	reverse	TCCCAAACACGACAACTCC
Cox4a	forward	ATTGGCAAGAGAGCCATTTCTAC
	reverse	CACGCCGATCAGCGTAAGT
Cox5b	forward	ACCCTAATCTAGTCCCGTCC
	reverse	CAGCCAAAACCAGATGACAG
Ndufa9	forward	GTCCGCTTTCGGGTTGTTAGA
	reverse	CCTCCTTTCCCGTGAGGTA
Sdha	forward	GGAACACTCCAAAAACAGACCT
	reverse	CCACCACTGGGTATTGAGTAGAA
Uqcrc2	forward	GCTAGAGCCATGAAGCTCCT
	reverse	TTCCCAAGTTGTTGGAGTCCT
Ppara	forward	CCACTACGGAGTTCACGCAT
	reverse	TTGCAGCTCCGATCACACTT
Glut2	forward	GCCCAGCAGTTCTCAGGAAT
	reverse	ACATGCCAATCATCCCGGTT
Gck	forward	AGACGAAACACCAGATGTATTCC
	reverse	GAAGCCCTTGGTCCAGTTGAG
